# In vitro fermentation characteristics of dietary fibers using fecal inocula from dogs treated with metronidazole

**DOI:** 10.1186/s42523-025-00459-z

**Published:** 2025-09-01

**Authors:** Sara E. Martini, Elizabeth L. Geary, Patrícia M. Oba, Laura L. Bauer, Ryan N. Dilger, Kelly S. Swanson

**Affiliations:** 1 Department of Animal Sciences, University of Illinois Urbana-Champaign, Urbana, IL 61801 USA; 2 Division of Nutritional Sciences, University of Illinois Urbana-Champaign, Urbana, IL 61801 USA; 3 Department of Veterinary Clinical Medicine, University of Illinois Urbana-Champaign, Urbana, IL 61801 USA

**Keywords:** Antibiotic, Canine microbiome, Gastrointestinal health, Fiber fermentation

## Abstract

Metronidazole is a potent antibiotic often prescribed to treat gastrointestinal enteropathies, but is known to induce loose stools, negatively alter the fecal microbiome, and affect fecal metabolites. Dietary intervention may aid in the recovery following antibiotic cessation, but little research has been conducted regarding the potential of fiber utilization for microbial recovery in canines. Using an in vitro fermentation assay, the objective of this study was to investigate the fermentation characteristics of dietary fibers using fecal inocula from dogs treated with metronidazole. Four healthy male beagles were fed a commercial kibble diet for 2 weeks, then administered metronidazole (20 mg/kg body weight twice a day) for 2 weeks. Fresh fecal samples were collected at weeks 2 and 4, stabilized in a 20% glycerol solution, and then frozen. For the in vitro fermentation experiment, feces from each time point (ABX−= pre-metronidazole collection; ABX+ = post-metronidazole collection) were thawed, diluted in an anaerobic diluting solution, and used to inoculate tubes. Tubes contained sterile medium and either cellulose, pectin, beet pulp, or chicory pulp fiber to test fermentation potential, with additional tubes used without fiber inclusion for blank corrections. At baseline (0 h) and after 6, 12, and 18 h of fermentation, pH, short-chain fatty acid (SCFA) production, and microbiota were measured. Data was analyzed within each fiber using the Mixed Models procedure of SAS version 9.4, with effects of antibiotic treatment, time and treatment*time interactions reported, accounting for the random effect across replicates. As expected, antibiotic administration had large effects on fiber fermentability characteristics, slowing pH reduction, lowering SCFA production, and altering SCFA molar ratios. Butyrate production was minimal among all fibers tested in ABX+ inocula tubes. Additionally, ABX+ inoculum lowered bacterial alpha diversity, affected bacterial beta diversity and the relative abundances of over 50 bacterial genera. Increased *Bifidobacterium* and *Lactobacillus* was observed in tubes containing ABX + inoculum (*P* < 0.0001) during pectin or beet pulp fermentation. Additionally, increased *Faecalibacterium, Streptococcus,* and *Bacteroides* was observed in tubes containing ABX- inoculum during chicory pulp fermentation (*P* < 0.0001). Beta diversity plots during beet pulp and chicory pulp fermentation demonstrated positive shifts toward ABX- inoculum tubes, but pectin fermentation did not yield the same shifts. The data presented here demonstrate that metronidazole administration can elicit unique responses to various fiber sources by reducing microbial diversity and negatively altering microbial fermentative activity (i.e., lower SCFA production). Both beet pulp and chicory pulp increased SCFA production and microbial diversity over time, with ABX+ inoculum tubes approaching that of ABX- inoculum tubes after 18 h of fermentation. More research is necessary but this data suggests that functional fibers promote microbial activity and recovery, and shed light on the potential effects of functional fibers in antibiotic-treated dogs.

**Supplementary Information:**

The online version contains supplementary material available at 10.1186/s42523-025-00459-z.

## Introduction

Several forms of canine chronic enteropathies exist, including food-responsive and antibiotic-responsive enteropathies, and are often a diagnosis of elimination of extra-intestinal factors [2, 17, 38, 56]. Attempts to treat chronic enteropathies may include diet alteration, steroid/immunosuppressive drugs, antibiotics, or fecal microbial transplantation treatments until clinical signs resolve. Antibiotics are among the first treatments for gastrointestinal infection or acute/subacute disease, with several classifications including penicillins, tetracyclines, quinolones, and macrolides. Antibiotics may be effective at treating some enteropathies but are known to induce rapid and negative side effects such as loose stools, altered microbial metabolism and reduced bacterial diversity. Repeated antibiotic use may also increase risk of antibiotic resistance. As recently addressed by Robbins et al. [51], 72% of antibiotics prescribed to over 1000 dogs (779) and cats (248) in the United States were bactericidal antibiotics (penicillins and fluoroquinolones; 1258/1724 total prescriptions), with 30% of dogs demonstrating antimicrobial drug resistance and 14% of dogs with multi-drug resistance. The results from that study showed relatively high antimicrobial resistance rates in dogs, suggesting that the usage of bactericidal antibiotics may be playing a prominent role in resistance development.

In companion animals, tylosin or metronidazole are commonly prescribed for GI related conditions. Metronidazole is a potent bactericidal agent and has previously been reported to reduce microbial populations and disrupt bacterial DNA synthesis and metabolism [14, 31, 47]. Previous studies in dogs, cats, humans, and rats have demonstrated that use of antibiotics can have effects beyond microbiota disruption (i.e., reduction in diversity, increased dysbiosis such as altering concentrations of microbial-derived metabolites [i.e., short-chain fatty acids (SCFA), bile acids, fatty acids and sterols] [4–7, 14, 33, 64]. SCFA (acetate, propionate, butyrate) are products of microbial fermentation from indigestible carbohydrates and largely produced in the large intestine [58]. SCFA are important to host health, as they serve as an energy source for host colonocytes, assist in lowering the luminal pH to restrict pathogenic bacteria growth, and improve gut barrier and integrity [22, 43, 46, 50]. Many commensal bacterial taxa are involved in SCFA production, including *Blautia, Faecalibacterium, Megamonas, Prevotella, Ruminococcus, Turicibacter,* and *Eubacterium* and are reduced with metronidazole use in dogs and cats [5–7, 28, 46, 60]. In individuals undergoing antibiotic treatment, restoration of these populations are crucial to reducing recovery time and maintaining long-term health.

Dietary fibers are plant-derived, non-digestible (soluble and insoluble) carbohydrates that have determined physiological benefits and can be classified based on properties such as solubility, fermentability, viscosity and water-holding capacity. Any combination of these properties can influence the rate and extent of fermentation, resulting in quantifiable outcomes such as SCFA production and changes to microbial abundances [26, 34]. Dietary fiber may be used to aid in microbial alterations, SCFA production, or modulating appetite, satiety or digestion and has a multitude of applications in diet formulation and veterinary medicine. Cellulose is an insoluble and non-viscous fiber that is reported to improve fecal quality, increase fecal bulk, and improve recovery in cases of acute diarrhea [10, 18, 30, 48]. On the other hand, pectin is a highly soluble and fermentable fiber, which can lead to increased production of SCFA and was shown to improve digestibility in dogs [55, 63]. While these are two common fibers in the pet food industry, fibers of other characteristics (i.e., moderately fermentable) might have other beneficial outcomes for companion animals.

In the pet food industry, a variety of dietary fibers may be selected, depending on their physiochemical properties and consequent effects on manufacturing processes, palatability, or host health. Beet pulp is a byproduct of sugar beet processing and is commonly used in pet food, as it is considered to have a nice balance of insoluble and soluble fibers, consisting of hemicelluloses, cellulose, and pectin fractions [18, 24]. Less commonly researched is the moderately fermentable fiber source, chicory pulp. In one study, chicory pulp was shown to have a chemical composition that was similar to beet pulp, with slightly higher organic matter (97.9% vs 92.8%), total dietary fiber (70.4% vs 63.0%), and insoluble fiber (53.9% vs 45.8%) on a dry matter basis [19]. In an in vitro fermentation assay using canine fecal inoculum, beet pulp and chicory pulp promoted the production of all SCFA but not as much as pectin [19]. Even though research on chicory pulp is limited, it has moderate fermentation potential and has promise as a functional fiber in pet food products.

To test the effects of nutritional interventions in animals, in vivo studies are often performed in the target host species but due to limitations (e.g., ethical concerns) with sample collection throughout the GI tract, in vitro fermentation systems may be utilized as an alternative approach. These in vitro fermentation systems can provide a low-cost, quick, and easy approach to study the GI microbiome by using a fecal sample collected from the host of interest [61]. In vitro fermentation systems are performed in a closed, temperature-controlled, and anaerobically-maintained environment but translation of in vitro to in vivo results are limited as these systems cannot account for physiological changes observed throughout the GI tract (e.g., changes in luminal pH and oxygen saturation, nutrient/metabolite absorption). However, these systems are highly reproducible and closely mimic the microbial composition and activity of microbes without ethical constraints [59]. Overall, these systems can allow for the monitoring of changes to microbial composition and activity, especially in response to specific substrates (e.g., dietary fibers) and have previously been used to test fermentation characteristics of dietary fibers in dog foods [19, 57].

The objective of this study was to investigate the fermentation characteristics of dietary fibers using fecal inocula from dogs treated with metronidazole. Based on their various physicochemical properties and fermentability levels, cellulose, pectin, beet pulp and chicory pulp were selected as fermentation substrates. Cellulose served as a negative control (low fermentation), pectin was the positive control (high fermentation), and beet pulp and chicory pulp were moderately fermentable test fibers. We hypothesized that fecal inoculum from dogs treated with antibiotics (ABX+) would have lower microbial diversity and would negatively influence fermentation rate and metabolite production. Of the substrates tested, we hypothesized that pectin would have the highest fermentability, followed by beet pulp, chicory pulp, and cellulose. Additionally, we predicted that pH would decrease and fermentation products (i.e., butyrate) would increase, while microbial composition would shift towards more SCFA producers (i.e., *Blautia, Bacteroides, Turicibacter, Lactobacillus*, *Bifidobacterium*) as the fermentability of fibers increased.

## Materials and methods

### Animals, diets, and experimental design

Four healthy adult male beagles (mean age = 1.63 ± 0.01 years; mean body weight = 9.15 ± 0.86 kg) were used to collect fresh fecal samples to be used for a source of inoculum. All dogs were housed individually in an environmentally-controlled facility at the University of Illinois Urbana-Champaign. Despite individual housing, dogs had constant access to toys and were allowed to socialize with humans and other dogs at least twice per week. Dogs had free access to fresh water at all times and were fed twice daily (8 a.m.; 3 p.m.). All dogs were fed a commercial diet (Pedigree Dog Chow; Pedigree Petfoods, McLean, VA) formulated to meet all nutrient recommendations for adult dogs at maintenance provided by the Association of American Feed Control Officials (AAFCO) [1] at a rate to maintain body weight. Food offered and refused was measured each day to calculate intake and any observations of vomiting or negative reactions were recorded. Dogs were weighed and body condition scores were assessed using a 9-point scale [36] once a week prior to the morning feeding throughout the study.

The study was 4 weeks in length. The study started with a 2-week baseline where all dogs consumed the diet only. After baseline, dogs received metronidazole (Metronidazole Compounded Oil Liquid Chicken Flavored; Chewy, Inc.; Boston, MA) at a dosage of 20 mg/kg orally twice daily (at mealtimes) for two weeks. Fresh fecal samples (within 15 min of defecation to limit oxygen exposure and ensure microbial activity was quickly preserved) were collected at the end of baseline (week 2; ABX−) and antibiotic administration (week 4; ABX+) and stabilized in a 20% glycerol solution in duplicate. Briefly, a 10 g fecal aliquot was collected in a 50 mL conical tube with 10 mL of a 20% glycerol solution [12]. Samples were then frozen at − 80 °C until the in vitro fermentation study was conducted.

### In vitro fermentation assay

On the day of the in vitro experiment, fecal samples were carefully thawed and heated to 39 °C using a water bath, pooled by treatment (ABX−= pre-metronidazole collection; ABX+ = post-metronidazole collection), and then diluted 1:4 (wt/vol) in anaerobic diluting solution and blended for 15 s in a Waring blender (Waring Products, Stamford, CT). Blended, diluted feces were filtered through 4 layers of cheesecloth (grade 10; 20 × 12 weave) and sealed in 125 mL serum bottles under a stream of CO_2_ to minimize exposure to oxygen. Sample and blank tubes were then aseptically inoculated with diluted feces and added to the medium (Table 1) as described by Bourquin et al. [9]. Briefly, this microbiological medium was formulated to include essential nutrients to sustain microbial viability and encourage microbial growth throughout the 18 h fermentation. Four mL of diluted feces were used to inoculate tubes containing 26 mL of semi-defined medium and one of the following fiber sources (300 mg/tube); cellulose (negative control), pectin (positive control), beet pulp, or chicory pulp.

**Table 1:** Composition of microbiological medium used in the in vitro experiment

Component	Amount
*Liquid solutions (mL/L)*	
Solution A^a^	330.0
Solution B^b^	330.0
Distilled water	296.0
Water-soluble vitamin mix^c^	20.0
Trace mineral solution^d^	10.0
Folate/biotin solution^e^	5.0
Riboflavin solution^f^	5.0
Hemin solution^g^	5.0
Resazurin^h^	1.0
Short-chain fatty acid mix^i^	0.4
*Solid chemicals, g in medium*	
Yeast	0.5
Trypticase	0.5
Na_2_CO_3_	4
Cysteine hydrochloride	0.5

^a^Composition (g/L): NaCl, 5.4; KH_2_PO_4_, 2.7; CaCl_2_·H_2_O, 0.18; MgCl_2_·6H_2_O, 0.12

MnCl_2_·4H_2_O, 0.06; CoCl_2_·6H_2_O, 0.06; (NH_4_)_2_SO_4_, 5.4

^b^Composition: K_2_HPO_4_, 2.7 g/L

^c^Composition (mg/L): thiamin hydrochloride,100; D-pantothenic acid, 100; niacin, 100; pyridoxine, 100; p-aminobenzoic acid, 5; vitamin B_12_, 0.25

^d^Composition (mg/L): EDTA (EDTA, disodium salt), 500; FeSO_4_·7H_2_O, 200; ZnSO_4_·7H_2_O, 10; MnCl_2_·4H_2_O, 3; H_3_PO_4_, 30; CoCl_2_·6H_2_O, 20; CuCl_2_·2H_2_O, 1; NiCl_2_·6H_2_O, 2; and Na_2_MoO_4_·2H_2_O, 3

^e^Composition (mg/L): folic acid, 10; D-biotin, 2; NH_4_HCO_3_, 100

^f^Hemin, 500 mg/L, in 10 mmol/L NaOH

^g^Composition: riboflavin, 10 mg/L, in 5 mmol/L of 4-(2-hydroxyethyl)piperazine-1-ethanesulfonic acid, N-(2-hydroxyethyl)piperazine-N′-(2-ethanesulfonic acid) (HEPES)

^h^Resazurin, 1 g/L, in distilled H_2_O

^i^Contained 250 µL/L each of *n*-valerate, isovalerate, isobutyrate, and *DL-α-*methylbutyrate

Triplicate tubes of each fibrous substrate were incubated at 39 °C for 0, 6, 12, or 18 h, with periodic mixing. At each time point, the incubation was stopped, and samples were processed immediately. For each time point, the pH of tube contents was measured using a pH meter. Samples to be analyzed (2 mL) for SCFA were mixed with 0.5 mL of 25% metaphosphoric acid and processed according to Erwin et al. [23] using a Hewlett-Packard (Avondale, PA) Model 5890A gas chromatograph equipped with a flame ionization detector on a column (1.8 m × 4 mm i.d.) packed with GP 10% SP-1200/1% H_3_P0_4_ on 80/100 chromosorb WAW (Supelco, Bellefonte, PA). The carrier gas was nitrogen, with a flow rate of 75 mL/min. The oven, injection port, and detector port temperatures were 125, 175, and 180 °C, respectively. Aliquots for microbial analyses were collected into sterile cryogenic vials and placed on dry ice until being transferred to a − 80 °C freezer where they were stored until analysis. Data were corrected by blank tube (inocula and media, but no fiber source) and baseline (0 h) sample production.

### DNA extraction and MiSeq illumina sequencing

Media collected after fermentation was centrifuged prior to extraction to improve DNA extraction techniques. Approximately 1–1.5 mL of fermentation media was transferred into a microtube and centrifuged 10,000×*g* at 4 °C for 10 min (Eppendorf Centrifuge 5424 R; Eppendorf Group, Hamburg, Germany). Supernatant was removed and the pellet was transferred to PowerBead tubes provided in the DNeasy PowerLyzer PowerSoil Kit (MoBio Laboratories, Carlsbad, CA) and DNA was extracted according to the manufacturer’s protocol. The supernatant then underwent bead beating using a vortex, followed by further centrifugation to purify DNA, then quantified using a Qubit® 3.0 Fluorometer (Life Technologies, Grand Island, NY). DNA quality was determined using an E-Gel Power Snap Electrophoresis Device (Invitrogen, Waltham, MA) on E-Gel EX 1% Agarose Gels. Concentration of extracted DNA was quantified using a Qubit 3.0 Fluorometer (Life Technologies, Grand Island, NY) and then submitted to the Roy J. Carver Biotechnology Center at the University of Illinois for Illumina sequencing with 16S rRNA gene amplicons that were generated using a Fluidigm Access Array (Fluidigm Corporation, South San Francisco, CA) in combination with Roche High Fidelity Fast Start Kit (Roche, Indianapolis, IN). The primers 515F (5′-GTGCCAGCMGCCGCGGTAA-3′) and 806R (5′-GGACTACHVGGGTWTCTAAT-3′) that target a 252 bp-fragment of the V4 region of the 16S rRNA gene were used for amplification (primers synthesized by IDT Corp., Coralville, IA) [13]. The CS1 forward tag and CS2 reverse tag were added according to the Fluidigm protocol. Quality of the amplicons were assessed using a Fragment Analyzer (Advanced Analytics, Ames, IA) to confirm amplicon regions and sizes. A DNA pool was generated by combining equimolar amounts of the amplicons from each sample. The pooled samples were then size selected on a 1% agarose E-gel (Life technologies, Grand Island, NY) and extracted using a Qiagen gel purification kit (Qiagen, Valencia, CA). Cleaned size-selected pooled products were run on an Agilent Bioanalyzer to confirm appropriate profile and average size. Illumina sequencing was then performed on a MiSeq using v3 reagents (Illumina Inc., San Diego, CA) at the Roy J. Carver Biotechnology Center at the University of Illinois.

### QIIME2 bioinformatics analysis

Illumina 16S rRNA gene amplicon sequencing produced a total of 483,994 sequences, with an average of 20,166 sequences per sample in tubes containing cellulose whereas pectin tubes contained the highest sequences at 555,887 (average 23,161 sequences per sample). Forward reads were trimmed using the FASTX-Toolkit (version 0.0.14), and sequences were analyzed using QIIME 2.0 version 2023.7 [13]. Raw sequence amplicons were imported into the QIIME2 package and analyzed by the DADA2 pipeline for quality control (QC value ≥ 20) [11]. After quality control, 364,656 reads were retained for cellulose, with the highest retention after quality control in pectin at 408,730 reads. All samples were rarefied with all samples retained after rarefication at the following reads: cellulose to 6,984 reads; pectin to 11,753 reads; beet pulp to 10,894 reads; chicory pulp to 5,178 reads. On average, 53.89% (45.97%, 69.01%, 68.76%, 31.82%, respectively) of features and 100% of the samples were retained after rarefication. Subsequent samples were assigned to taxonomic groups with the SILVA database (SILVA 138; 99% OTU from 515F/806R region of sequences, with the QIIME2 classifier trained on 515F/806R V4 region of 16S) [8, 49, 52]. The rarefied samples were used for alpha diversity and beta diversity. Principal coordinates analysis was performed using weighted and unweighted unique fraction metric (UniFrac) distances [37].

### Statistical analysis

Data was blank-corrected and analyzed using the Mixed Models procedure of SAS version 9.4 (SAS Institute Inc., Cary, NC), with antibiotic treatment and time as fixed effects and random effect of each replicate, within each fiber. Normality was tested using the UNIVARIATE procedure of SAS. If data did not meet normality, data were analyzed using npa1rway procedures and Wilcoxon statistics were used to determine significance. Mean change from 0 h differences due to antibiotic (ABX-, ABX+), time (Δ6 h, Δ12 h, Δ18 h), and antibiotic*time were determined using a Fisher-protected least significant difference with a Tukey adjustment to control for experiment-wise error. Statistical significance was set at *P* < 0.05, with tendencies having *P* > 0.05 and < 0.10.

## Results

### Baseline measures

As a positive control, highly fermentable pectin was used and cellulose was selected to serve as a negative control due to its low fermentative potential. Selected moderately fermentable fibers (beet pulp and chicory pulp) were utilized as test fibers in the present study. Baseline (0 h) pH and SCFA concentrations for tubes containing pectin, beet pulp, chicory pulp, and cellulose are presented in Supplemental Table 1. Only pectin tubes were affected by inoculum, with ABX + inoculum demonstrating higher pH measures (*P* < 0.05) at baseline (0 h). Pectin, beet pulp and cellulose tubes containing ABX+ inoculum had lower propionate concentrations (*P* < 0.05) at baseline compared to tubes containing ABX- inoculum. Remaining SCFA (acetate, propionate) concentrations were not different at baseline among any fiber.

In tubes containing ABX- or ABX + inoculum, all baseline blank-corrected bacterial phyla and genera relative abundances (% of sequences) are presented in Supplemental Table 2, with few differences were observed. Phyla Bacteroidota and Fusobacteriota, were higher in tubes containing ABX- inoculum while Proteobacteria was higher in tubes containing ABX + inoculum (*P* < 0.05). At baseline (0 h), tubes containing ABX- inoculum had higher (*P* < 0.05) *Muribaculaceae*, *Parabacteroides, Prevotella,* Lachnospiraceae unclassified, *Lachnospiraceae_NK4A136_group, Megamonas,* Ruminococcaceae unclassified, *Terrisporobacter*, and *Fusobacterium*. In baseline (0 h) tubes containing ABX + inoculum, *Coriobacteriaceae_UCG-002, Parvibacter*, *[Eubacterium]_brachy_group*, *[Ruminococcus]_torques_group, Allobaculum, Anaeroplasma, Erysipelotrichaceae_UCG-003, Faecalibaculum, Lachnoclostridium, Turicibacter, Escherichia-Shigella, Parasutterella,* and *Proteus* were higher (*P* < 0.05).

### Pectin fermentation

**Fig. 1:**
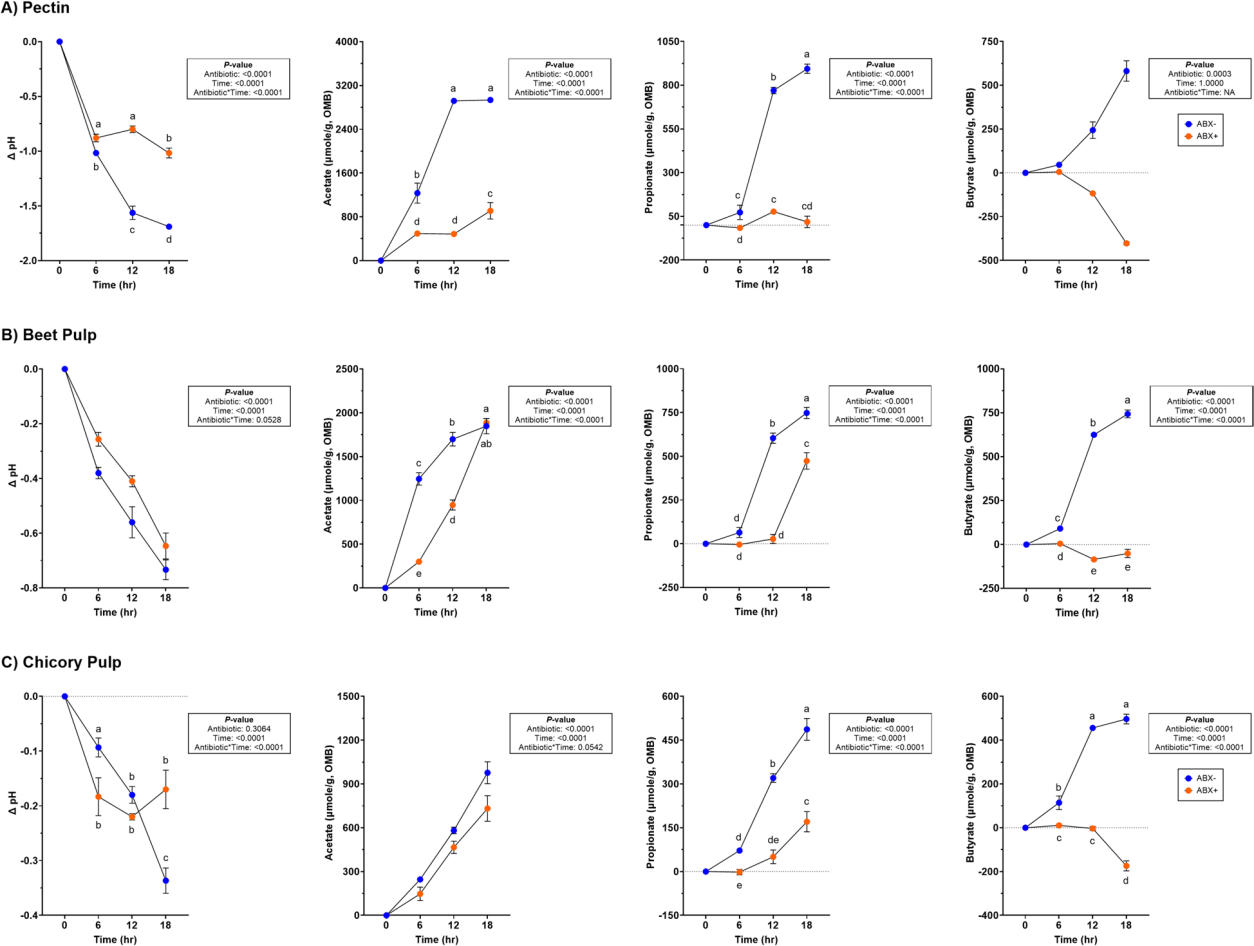
Measures of pH and SCFA concentrations (μmole/g, OMB) in tubes containing (**A**) pectin, (**B**) beet pulp, and (**C**) chicory pulp. Data are presented as change from baseline (0 h) least square means ± SEM. Samples from tubes containing pre-antibiotic collected inoculum (ABX−) are blue and samples from tubes containing post-antibiotic collected inoculum (ABX+) are orange

Significantly lower pH measures (*P* < 0.0001) were observed in tubes containing ABX- inoculum and increased concentrations of all SCFA (*P* < 0.001; Supplemental Table 3; Fig. 1). Butyrate concentrations significantly declined in tubes containing ABX + inoculum (*P* < 0.001).

**Fig. 2:**
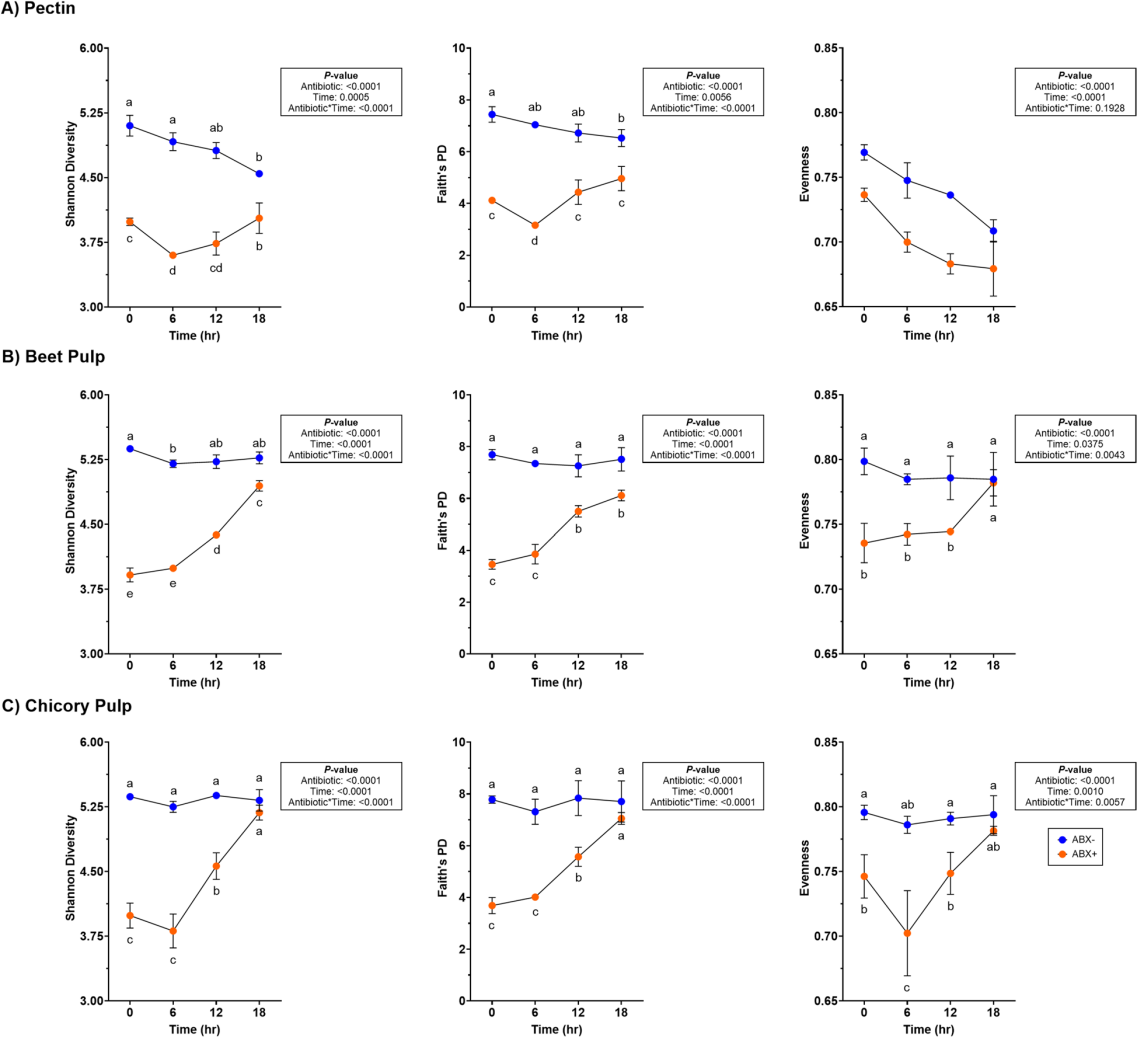
Alpha diversity measures in tubes containing (**A**) pectin, (**B**) beet pulp, and (**C**) chicory pulp. Data are presented as least square means ± SEM. Samples from tubes containing pre-antibiotic collected inoculum (ABX−) are blue and samples from tubes containing post-antibiotic collected inoculum (ABX+) are orange

**Fig. 3:**
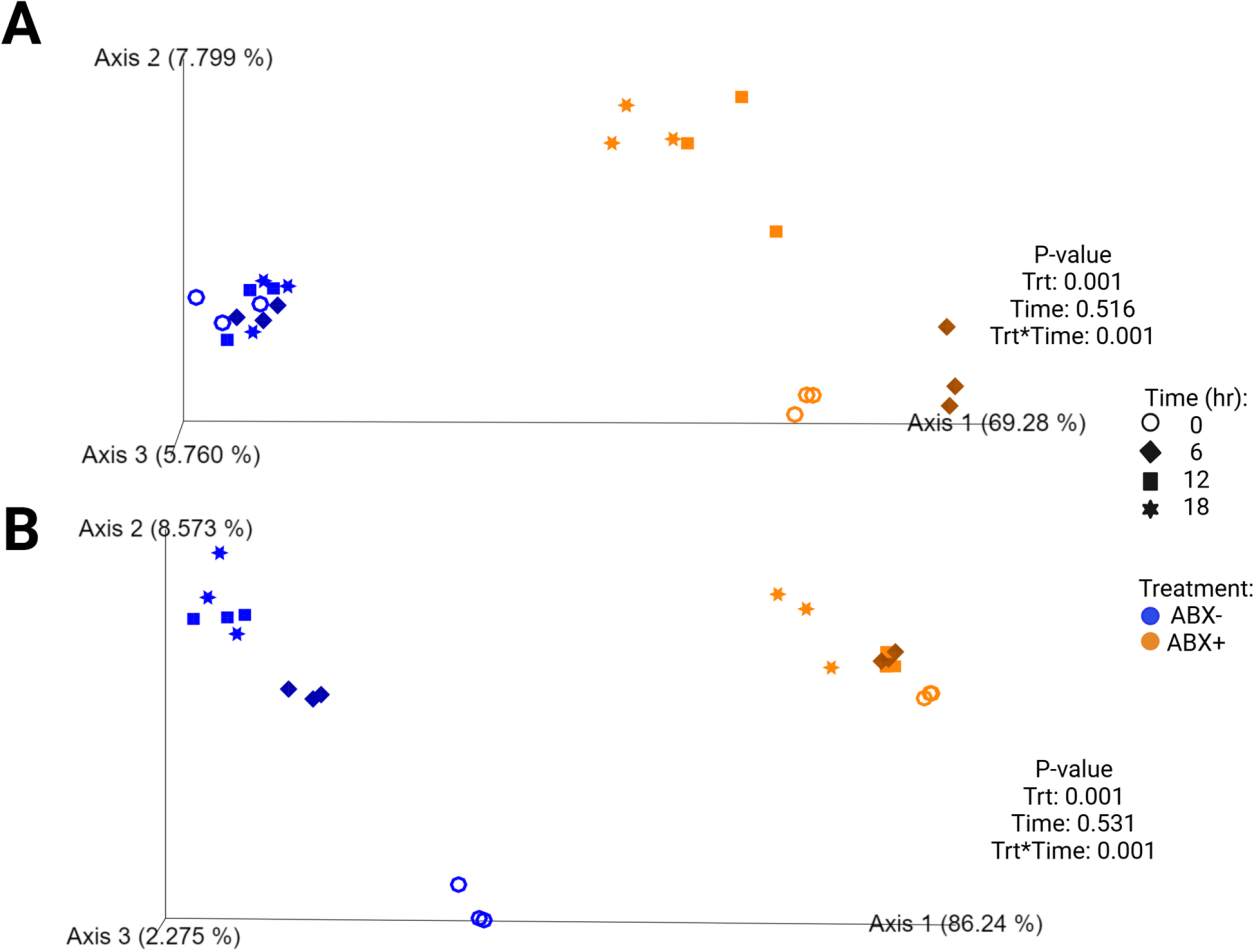
Unweighted (**A**) and weighted (**B**) bacterial beta diversity plots of in vitro fermentation tubes containing pectin. Samples from tubes containing pre-antibiotic collected inoculum (ABX−) are blue and samples from tubes containing post-antibiotic collected inoculum (ABX+) are orange

Fecal bacterial alpha diversity measures decreased with time (*P* < 0.0001) across all alpha diversity parameters measured in tubes containing ABX- inoculum (Fig. 2; Supplemental Table 4). Only Shannon diversity and Faith’s Phylogenetic Diversity (PD) increased with time (*P* < 0.01) but evenness continued to decrease (*P* < 0.0001) during fermentation in tubes containing ABX + inoculum. Beta diversity plots demonstrated how pectin fermentation shifted the fecal microbiota populations (Fig. 3). Both unweighted and weighted diversity plots show clustering by antibiotic treatment, with all ABX- clustering together across all time points. Tubes containing ABX + inoculum separated by time point and demonstrated shifts toward tubes containing ABX- inoculum.

In tubes containing pectin, all change from baseline relative abundances presented in Supplemental Table 5. Actinobacteria and Firmicutes were increased (*P* < 0.0001) in tubes containing ABX + inoculum and Bacteroidota was increased (*P* < 0.0001) in tubes containing ABX- inoculum. Fusobacteria decreased over time (*P* < 0.0001) and Proteobacteria decreased more in ABX + (*P* = 0.0003). Pectin fermentation increased *Bifidobacterium, Enterococcus, Lactobacillus* and *Streptococcus* in tubes containing ABX + inoculum (*P* < 0.01) whereas *Bacteroides* and *Faecalibacterium* increased more in tubes containing ABX- inoculum (*P* < 0.001). With pectin fermentation, *Blautia* and *Fusobacterium* decreased over time (*P* < 0.0001) but increased after 18-h in tubes containing ABX- inoculum (*P* < 0.0001).

### Beet pulp fermentation

No significant antibiotic*time was observed for pH measures but tubes containing ABX- inoculum had larger pH reductions (*P* < 0.0001; Fig. 1; Supplemental Table 3). All SCFA concentrations were increased with time, with ABX- demonstrating increased SCFA concentrations (*P* < 0.0001). Propionate concentrations increased by 18-h but were lower in tubes containing ABX + inoculum (*P* < 0.0001). Butyrate production was significantly affected and decreased in tubes containing ABX + inoculum across all time points (*P* < 0.0001).

**Fig. 4:**
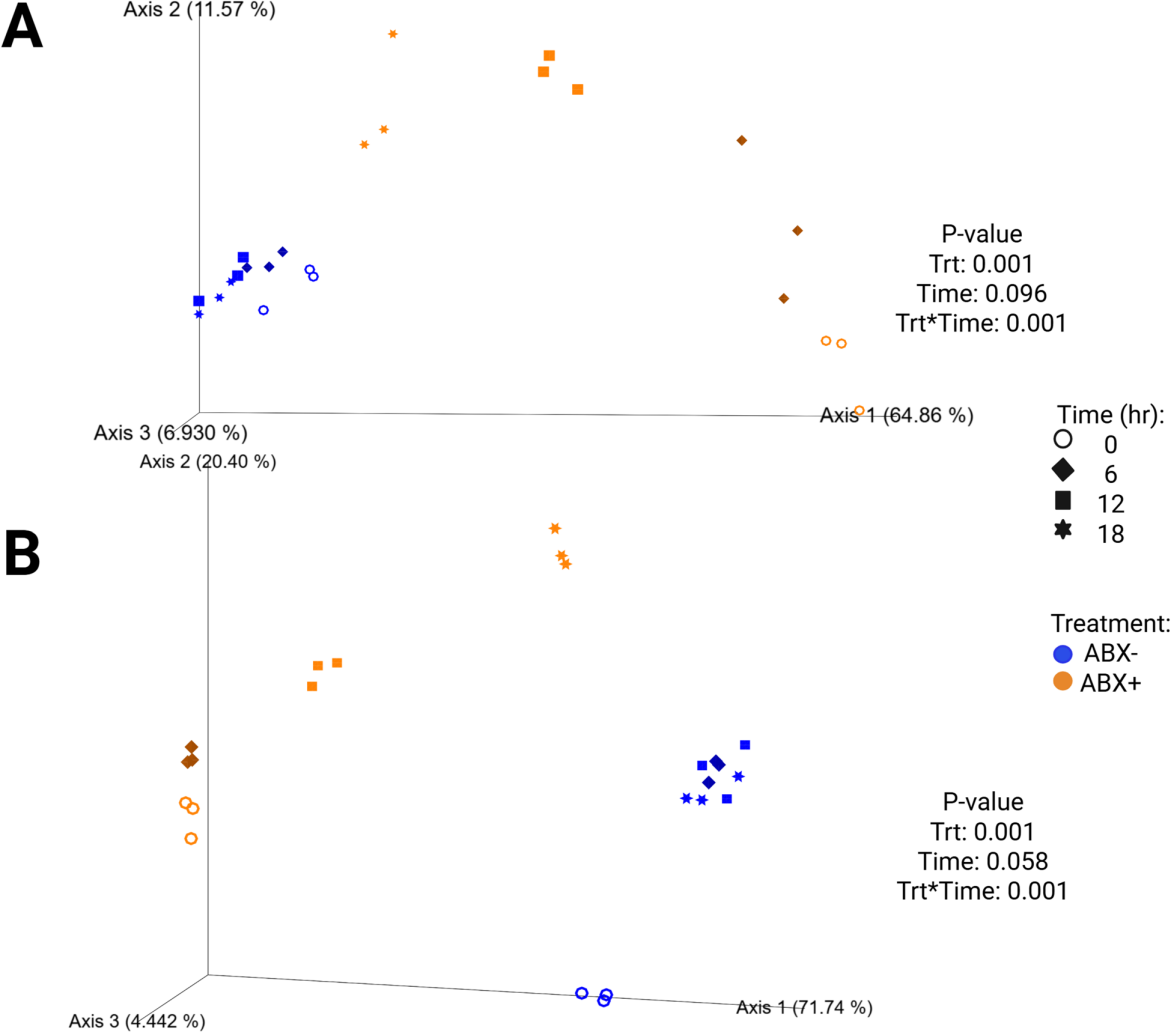
Unweighted (**A**) and weighted (**B**) bacterial beta diversity plots of in vitro fermentation tubes containing beet pulp. Samples from tubes containing pre-antibiotic collected inoculum (ABX−) are blue and samples from tubes containing post-antibiotic collected inoculum (ABX+) are orange

Alpha diversity measures of beet pulp fermentation are presented in Fig. 2 (Supplemental Table 4). All diversity parameters increased with time (*P* < 0.05). Shannon diversity and Faith’s PD were lower in tubes containing ABX + inoculum by 18-h (*P* < 0.0001) but not different from tubes containing ABX- inoculum for evenness measures at the end of fermentation. Unweighted and weighted beta diversity plots demonstrate significant clustering by antibiotic treatment (*P* = 0.001) with tendencies (*P* < 0.10) to separate by time (Fig. 4). Tubes containing ABX + inoculum showed diversity shifts towards tubes containing ABX- inoculum by 18-h.

**Fig. 5:**
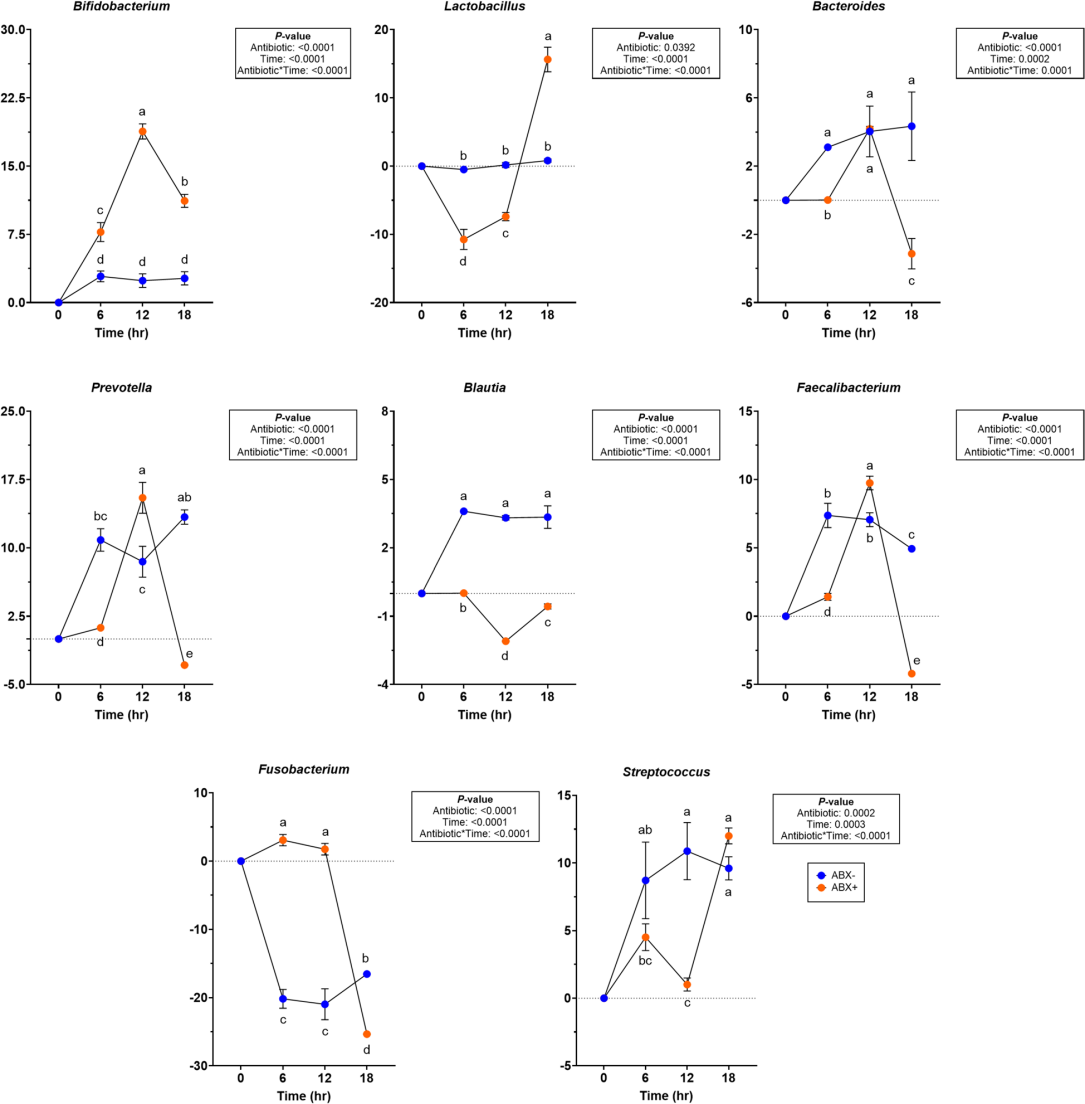
Changes to relative abundances (% of sequences) during beet pulp in vitro fermentation using fecal inocula collected from dogs before (ABX−) and after (ABX+) metronidazole administration. Data are presented as change from baseline (0 h) least square means ± SEM. Samples from tubes containing pre-antibiotic collected inoculum (ABX−) are blue and samples from tubes containing post-antibiotic collected inoculum (ABX+) are orange

Significant changes were observed on phyla and genera levels (Supplemental Table 6). During beet pulp fermentation, *Bifidobacterium* and *Lactobacillus* abundances increased significantly in tubes containing ABX + inoculum (*P* < 0.0001; Fig. 5). *Bacteroides, Prevotella, Blautia, Faecalibacterium,* and *Fusobacterium* abundances were reduced more in tubes containing ABX + inoculum (*P* < 0.0001). *Streptococcus* abundances in tubes containing ABX + inoculum, were reduced after 12-h of fermentation (*P* < 0.0001) but increased and were not different with tubes containing ABX- inoculum by the end of fermentation.

### Chicory pulp fermentation

Overall pH during fermentation decreased in both treatments, with larger reductions observed in tubes containing ABX- inoculum after 18-h and tubes containing ABX + inoculum were not different throughout fermentation (*P* < 0.0001; Fig. 1; Supplemental Table 3). All SCFA concentrations increased in tubes containing ABX- inoculum (*P* < 0.0001). Tubes containing ABX + inoculum had less propionate and butyrate production, with depletion of butyrate concentrations by 18-h (*P* < 0.0001).

**Fig. 6:**
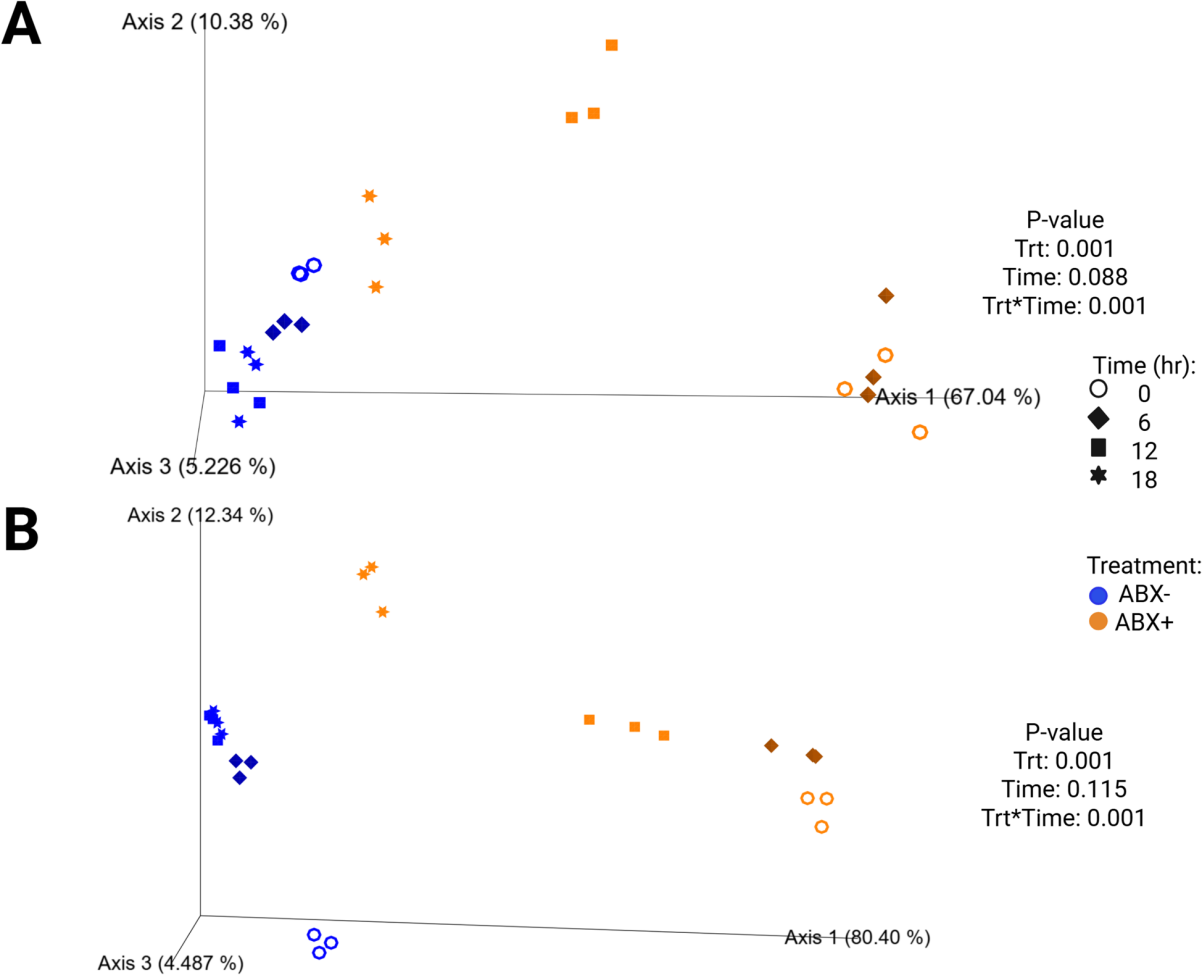
Unweighted (**A**) and weighted (**B**) bacterial beta diversity plots of in vitro fermentation tubes containing chicory pulp. Samples from tubes containing pre-antibiotic collected inoculum (ABX−) are blue and samples from tubes containing post-antibiotic collected inoculum (ABX+) are orange

Measures of alpha diversity in tubes used for chicory pulp fermentation are presented in Fig. 2 (Supplemental Table 4). All alpha diversity measures were higher in tubes containing ABX- inoculum and were the lowest in tubes containing ABX + inoculum at 0-h (*P* < 0.0001). By 18-h, tubes containing ABX + inoculum increased and were not different from tubes containing ABX- inoculum (*P* < 0.01). Shifts in the bacterial beta diversity of in vitro fermentation tubes during chicory pulp fermentation are presented in Fig. 6. Strong clustering is observed within antibiotic treatment of both unweighted and weighted plots. The unweighted plot demonstrate minimal shifts in chicory pulp fermentation in tubes containing ABX- inoculum. In the weighted plot, significant shifts away from 0-h was observed in tubes containing ABX- inoculum but greater shifts were observed in tubes containing ABX + inoculum.

**Fig. 7:**
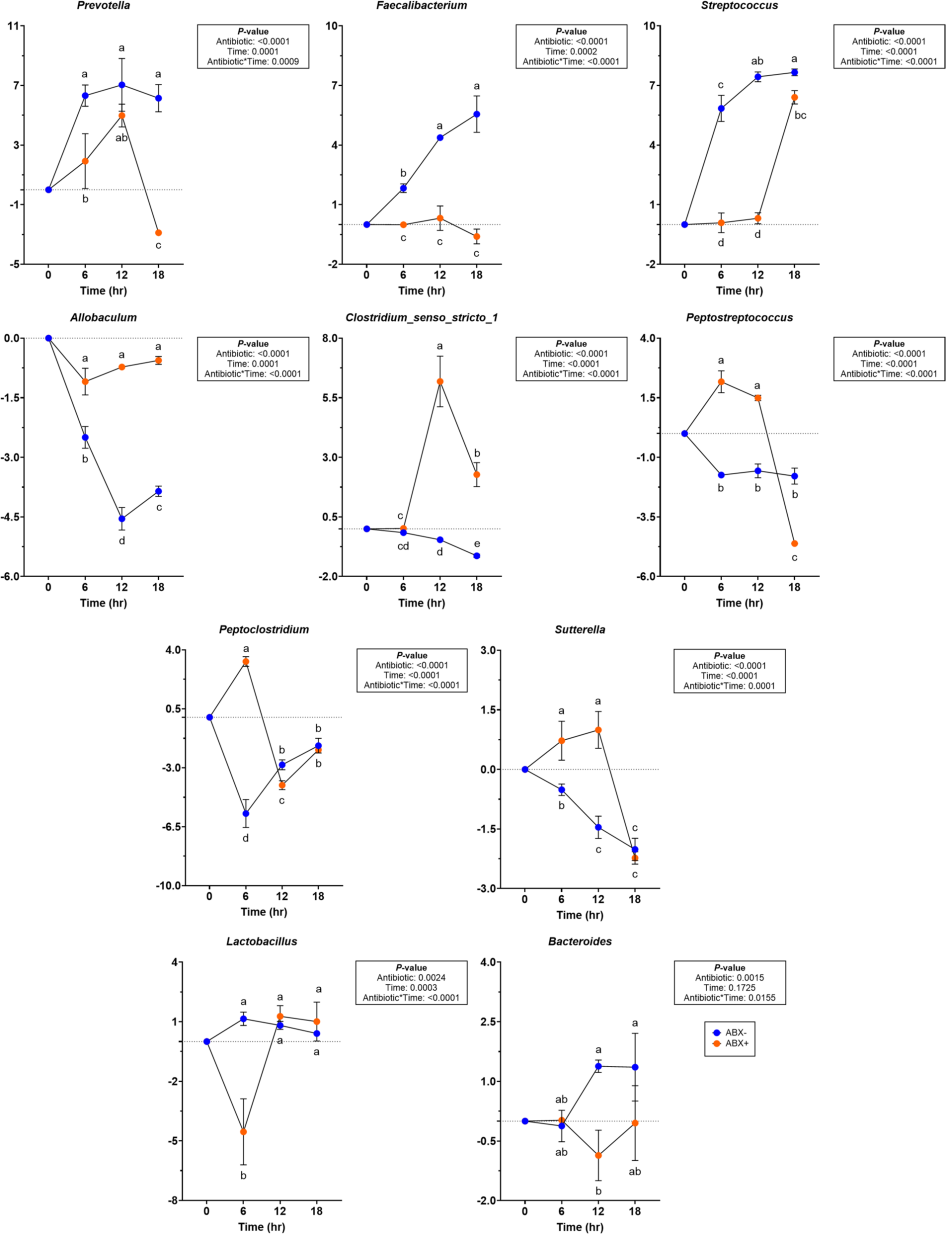
Changes to relative abundances (% of sequences) during chicory pulp in vitro fermentation using fecal inocula collected from dogs before (ABX-) and after (ABX+) metronidazole administration. Data are presented as change from baseline (0 h) least square means ± SEM. Samples from tubes containing pre-antibiotic collected inoculum (ABX−) are blue and samples from tubes containing post-antibiotic collected inoculum (ABX+) are orange

At the phyla level, changes to Firmicutes abundances were not observed (Supplemental Table 7). Actinobacteria and Bacteroidota increased more in tubes containing ABX- inoculum (*P* < 0.0001). Fusobacteria had greater reductions in tubes containing ABX- inoculum and Proteobacteria had greater reduction in tubes containing ABX + inoculum (*P* < 0.01). Minor changes in abundances were observed within genera (Fig. 7; Supplemental Table 7). In tubes containing ABX- inoculum, *Blautia* were increased (*P* < 0.0001) and had significant reductions (*P* = 0.0003) in *Fusobacterium*. *Bifidobacterium* and *Escherichia-Shigella* were significantly reduced in tubes containing ABX + inoculum (*P* < 0.001). *Enterococcus* abundances increased significantly more (*P* < 0.0001) in tubes containing ABX + inoculum. *Prevotella, Faecalibacterium* and *Streptococcus* were higher by 18-h (*P* < 0.001) in tubes containing ABX- inoculum (Fig. 7). In tubes containing ABX- inoculum, *Allobaculum* and *Clostridium_senso_stricto_1* were significantly reduced (*P* < 0.0001) and *Peptostreptococcus* had greater reduction (*P* < 0.0001) in tubes containing ABX+ inoculum. *Peptoclostridium* and *Sutterella* increased while *Lactobacillus* decrease in tubes containing ABX+ inoculum but were not different from tubes containing ABX- inoculum after 18-h (*P* < 0.0001). *Bacteroides* increased significantly in tubes containing ABX- inoculum by 12-h (*P* = 0.0155) but was not different from tubes containing ABX + inoculum by 18-h.

### Cellulose fermentation

During fermentation, no differences were observed in pH or butyrate concentrations (*P* > 0.05; Supplemental Table 3). Acetate and propionate concentrations, however, were affected by a antibiotic*time interaction (*P* < 0.05), with tubes containing ABX- inoculum having the highest productions by the end of fermentation.

Alpha diversity measures of tubes containing cellulose is presented in Supplemental Table 4. Tubes containing ABX- inoculum were not different with time for any diversity parameters measured. Tubes containing ABX + inoculum increased with time (*P* < 0.05), and were different from tubes containing ABX- inoculum for Shannon diversity or evenness at 18-h. Beta diversity plots reflecting fecal bacterial shifts throughout fermentation are presented in Supplemental Fig. 1. Both unweighted and weighted plots demonstrate separate clustering by metronidazole treatment and by clustering within time points during fermentation.

Changes to bacterial phyla and genera observed during fermentation presented in Supplemental Table 8. Actinobacteridota increased significantly (*P* < 0.05) by 6-h of fermentation but not significantly different for the remainder of fermentation. *Bifidobacterium, Collinsella, Dubosiella, Lactobacillus* and *Escherichia-Shigella* abundances increased significantly in tubes with ABX + inoculum by 18-h (*P* < 0.0001). *Bacteroides* were increased in tubes containing ABX + inoculum at 6-h and 12-h but significantly decreased (*P* < 0.0001) after 18-h of fermentation, whereas tubes containing ABX- inoculum were not affected. *Faecalibacterium* abundances were increased by 12-h but declined by 18-h (*P* < 0.0001) in tubes containing ABX + inoculum. *Streptococcus* abundances were highest at 18-h of fermentation (*P* < 0.01) but not affected by metronidazole.

## Discussion

As previously mentioned, antibiotics are one of several therapeutic options in cases of GI disease and many studies demonstrate microbial shifts and metabolite alterations as a result of administration in dogs [6, 7, 14, 31, 47]. To increase abundances of commensal and SCFA-producing bacteria (i.e., *Blautia, Faecalibacterium*), dietary intervention or supplementation may be useful via incorporation of functional ingredients. Inclusion of dietary fiber (i.e., cellulose, pectin, beet pulp, chicory pulp) or prebiotics (i.e., inulin, lactulose, fructo- or galacto- oligosaccharides) into a treatment plan may be an effective strategy as many of these have been studied for their influence on microbiota and SCFA production. However, a recent review conducted by Wilson and Swanson [62] suggests that the inclusion of prebiotics may be more useful in prevention of gastrointestinal diseases rather than treatment due to the selectivity of SCFA-producing bacteria. Previous studies have demonstrated that dietary fibers, including both soluble and insoluble fractions, are capable of increasing SCFA concentrations in canine feces [18, 20, 21, 25, 41]. Differences measured in bacterial metabolic outcomes presented can be attributed to the diversity in fiber characteristics (e.g., solubility, fermentability). When combining these outcomes with advanced microbial technologies and a greater understanding of microbial gene functions, this microbial response can provide further insight into how various fibers can be utilized by microbes in ways that the host species cannot [29, 39].

The pH of the GI environment is one of many factors that can influence microbial presence and function. Changes to pH can alter microbial composition, with lower measures demonstrating limited or reduced growth of pH-sensitive bacteria (i.e., *Streptococcus* and *Veillonella*) [32]. Measured in the current study through fermentation media, 18-h of fermentation demonstrated greater pH changes in tubes containing pre-metronidazole collected inoculum with pectin and chicory pulp fermentation whereas post-metronidazole collected inoculum tended to follow similar patterns but did not achieve statistical significance. In a 16-h in vitro fermentation study conducted by de Godoy et al. [19], more fermentable fibers (i.e., pectin and short-chain fructooligosaccharides) had the greatest pH reduction compared to pelletized cellulose. Similar results were observed in the present study as pH was not influenced by antibiotic treatment in cellulose fermentation but was significantly reduced in pectin and beet pulp fermentation, with less changes observed with chicory pulp.

As previously stated, pH can not only influence microbial presence, but it can cause metabolic disruption and production of SCFA [32] and has previously been observed in dogs treated with metronidazole [6, 7]. A previous experiment using in vitro fermentation methods with canine inoculum demonstrated that highly fermentable fibers (i.e., citrus pectin, fructooligosaccharides, guar gum, lactulose) promoted the highest organic matter disappearance and SCFA production, demonstrating more efficient utilization compared to cellulose-rich fibers (i.e., oat fiber and Solka-Floc) [57]. Consistent with findings in the present study, pectin, beet pulp and chicory pulp demonstrated increased SCFA production. However, each fiber substrate yielded variable fermentation patterns and altered pH measures, when inoculated with post-metronidazole samples. SCFA production was greatly limited in tubes with post-metronidazole collected inoculum, with cessation of butyrate production in pectin, beet pulp and chicory pulp treatments. Beet pulp in tubes containing post-metronidazole collected inoculum was successful in promoting acetate production to similar concentrations in tubes containing pre-metronidazole collected inoculum, neither of the other fermentable fibers were successful in modulating SCFA production to the same degree. Overall, fibers of higher fermentability characteristics showed more variation in fermentation response in post-metronidazole collected inoculum.

In agreement with results observed in the current study, the use of dietary fibers in vitro increased counts of beneficial bacteria including *Bifidobacterium, Lactobacillus, Bacteroides, Prevotella,* and *Faecalibacterium,* and reduced counts of *Fusobacterium* [15, 16, 27, 35, 40, 42, 44–46, 54]. While *Streptococcus* abundances increased during fermentation regardless of metronidazole treatment, literature regarding this genera has considered this genera to be opportunistic in nature and associated negative outcomes with increased abundances in canine GI disease [3, 53].

Evaluating alpha diversity measures are useful for quantifying bacterial richness and evenness, which increased throughout fermentation in post-metronidazole collected inoculum during beet pulp and chicory pulp fermentation. In general, increases in diversity are regarded as beneficial outcomes, demonstrating a more diverse microbiota. Interestingly, pectin fermentation demonstrated a decline in all alpha diversity measures in pre-metronidazole collected inoculum, whereas post-metronidazole collected inoculum demonstrated increases, but did not achieve near pre-metronidazole initial diversity measures after 18-h. This was an interesting outcome as it suggested increased microbial diversity, similar to beet pulp and chicory pulp, but only after metronidazole usage. Also, this outcome suggests potential favoring of few specific bacteria which contributes to less overall diversity, as shaped by substrate availability [39]. Fruit fibers used in the animal industry have high pectin content [18] but is known to be rapidly fermented upon entering the GI tract. While dietary fibers are typically heterogeneous blends in diets, pectin inclusion in metronidazole recovery might be beneficial in a limited quantities, with higher inclusion of beet pulp and chicory pulp.

Limitations to using in vitro fermentation assays as a model for mimicking a host GI system should be addressed for the present study. First, in vitro fermentation assay is temperature-controlled and anaerobically maintained; however, the closed-system does not correct for host GI secretions (i.e., host enzymes, mucus, bile acids) or for in vivo SCFA production prior to sample collection and storage. Also, the physiological properties of the host GI tract (i.e., oxygen concentration, luminal pH) vary greatly and are not replicated using the in vitro model. These properties can influence microbial response and interactions with other commensal species, and ultimately how secondary microbial metabolism is performed. While the microbiological medium is formulated to include essential nutrients microbes need to survive the transition from living host to in vitro closed systems, this may also influence microbial activity and can contribute to a lag phase of growth during the transition. Additionally, only four time points (0, 6, 12, 18 h) were sampled to measure outcomes, while more frequent sampling could have provided a more comprehensive view of fermentation kinetics and quantified potential lag phase functions. Applying this data to the canine population may be limited as only four animals selected for this study were of similar backgrounds. Using a more diverse population of canines could have yielded different outcomes. Lastly, the four test fibers selected are common to the pet food industry and only represent a small fraction of what may be used in commercial diets. Expanding the panel of test fibers with greater variation in fiber characteristics (i.e., solubility, fermentability) may have demonstrated additional unique outcomes applicable to personalized or targeted formulations for pet food or supplemental products targeted toward beneficial microbial shifts.

In summary, metronidazole remains to be a potent antimicrobial with strong ability to reduce microbial metabolism and diversities as demonstrated by the quantification of SCFA fermentation products, microbial abundances, and diversity measures. By providing in vitro fermentation tubes with fibers of varying properties (i.e., fermentation potential, solubility), increased alpha diversity and microbial abundances of beneficial bacteria such as *Bacteroides, Bifidobacterium, Faecalibacterium,* and *Blautia* were observed in tubes with post-metronidazole inoculum, which could be indicative of increased microbial recovery and presence. Additionally, increased production of acetate and propionate were observed in tubes provided pectin, beet pulp, and chicory pulp, demonstrating positive fermentation potential but was less extensive with post-metronidazole inocula. From these presented results, we can conclude that while antibiotics may be a necessary treatment plan in clinical settings, additional consideration should be given to their effects beyond the desired intention, such as their influence on bacterial metabolism, especially in regard to diet consumption and consequently, dietary fiber fermentation patterns.

## Supplementary Information

**Additional file 1**.

## Data Availability

A supplementary data file is available online for download that includes Supplemental Tables 1-8 and Supplemental Fig. 1. All sequence data used for analysis are available at the NCBI sequence read archive under BioProject PRJNA1260019 (https://www.ncbi.nlm.nih.gov/bioproject/PRJNA1260019).

## Abbreviations

ABX−: Pre-antibiotic collected inoculum
ABX+: Post-antibiotic collected inoculum
GI: Gastrointestinal
SCFA: Short-chain fatty acid
