# Supplementary material for: In vitro fermentation characteristics of dietary fibers using fecal inocula from dogs treated with metronidazole
Source: Anim Microbiome. 2025 Sep 1;7:93. doi: 10.1186/s42523-025-00459-z (PMC12403923; doi:10.1186/s42523-025-00459-z)

# **Supplemental Table 1.** Baseline (0 hr) pH and SCFA concentrations (μmole/g, OMB) of tubes containing pectin, beet pulp, chicory pulp, or cellulose

| Fiber |  | ABX-^1^ | ABX+ | SEM^2^ | p-value |
| --- | --- | --- | --- | --- | --- |
| Pectin | pH | 6.90^b^ | 7.04^a^ | 0.031 | 0.0145 |
|  | Acetate | 30.87 | 32.80 | 9.062 | 0.8139 |
|  | Propionate | 13.07^a^ | 3.51^b^ | 3.352 | 0.0454 |
|  | Butyrate | 4.54 | 2.41 | 2.246 | 0.4964 |
| Beet pulp | pH | 6.91 | 6.93 | 0.012 | 0.3701 |
|  | Acetate | 125.96 | 130.18 | 5.082 | 0.5889 |
|  | Propionate | 17.09^a^ | 6.58^b^ | 2.123 | 0.0121 |
|  | Butyrate | 4.01 | 3.13 | 2.044 | 0.4338 |
| Chicory pulp | pH | 6.94 | 6.95 | 0.009 | 0.4778 |
|  | Acetate | 44.23 | 51.97 | 7.168 | 0.4873 |
|  | Propionate | 16.25^a^ | 4.50^b^ | 1.924 | 0.0125 |
|  | Butyrate | 5.94 | 5.22 | 1.111 | 0.6735 |
| Cellulose | pH | 6.97 | 6.99 | 0.002 | 0.0377 |
|  | Acetate | 16.05 | 23.31 | 4.756 | 0.1712 |
|  | Propionate | 14.21^a^ | 4.09^b^ | 1.021 | 0.0139 |
|  | Butyrate | 3.53 | 3.40 | 1.066 | 0.9257 |

^1^ABX- = samples collected from dogs prior to antibiotic administration; ABX+ = samples collected after receiving metronidazole (20 mg/kg body weight twice daily) for two wk.
^2^SEM = pooled standard errors of the means.
^ab^Mean values within a row with unlike superscript letters differ using parametric analysis (P<0.05).

# **Supplemental Table 2.** Blank-corrected bacterial phyla and genera relative abundances (% of sequences) at baseline (0 hr) of tubes containing ABX- or ABX+ inoculum

| Phyla | Genera | ABX-^1^ | ABX+ | SEM^2^ | p-value |  |
| --- | --- | --- | --- | --- | --- | --- |
| Actinobacteridota | | 0.05 | 0.48 | 0.915 | 0.5579 |  |
|  | *Adlercreutzia* | 0.01 | 0.00 | 0.011 | 0.3383 |  |
|  | *Bifidobacterium* | 0.31 | 0.61 | 0.788 | 0.6490 |  |
|  | *Collinsella* | -0.15 | -0.13 | 0.131 | 0.9025 |  |
|  | *Coriobacteriaceae_UCG-002* | -0.06^y^ | 0.00^z^ | 0.019 | 0.0263 |  |
|  | *Parvibacter* | -0.03^y^ | 0.00^z^ | 0.009 | 0.0262 |  |
|  | *Slackia* | -0.02 | 0.00 | 0.007 | 0.1414 |  |
| Bacteroidota | | 0.65^z^ | 0.00^y^ | 0.260 | 0.0375 |  |
|  | *Alloprevotella* | 0.05 | 0.02 | 0.015 | 0.1648 |  |
|  | *Bacteroides* | 0.02 | 0.00 | 0.157 | 0.1547 |  |
|  | *Muribaculaceae* | 0.30^z^ | 0.00^y^ | 0.066 | 0.0031 |  |
|  | *Parabacteroides* | 0.03^z^ | 0.00^y^ | 0.005 | 0.0031 |  |
|  | *Prevotella* | 0.20^z^ | -0.01^y^ | 0.061 | 0.0036 |  |
|  | *Prevotellaceae_Ga6A1_group* | 0.05 | 0.00 | 0.016 | 0.1387 |  |
| Firmicutes | | -2.00 | -2.29 | 1.039 | 0.8096 |  |
|  | *[Eubacterium]_brachy_group* | -0.05^y^ | 0.00^z^ | 0.009 | 0.0263 |  |
|  | *[Ruminococcus]_gauvreauii_group* | 0.01 | 0.00 | 0.017 | 0.7346 |  |
|  | *[Ruminococcus]_gnavus_group* | -0.09 | 0.01 | 0.049 | 0.1256 |  |
|  | *[Ruminococcus]_torques_group* | -0.14^y^ | 0.00^z^ | 0.045 | 0.0263 |  |
|  | *Allobaculum* | -0.64^b^ | 0.16^a^ | 0.198 | 0.0044 |  |
|  | *Anaeroplasma* | 0.01^y^ | 0.04^z^ | 0.301 | 0.0105 |  |
|  | *Blautia* | 0.10 | 0.00 | 0.112 | 0.4591 |  |
|  | *Butyricicoccus* | -0.01 | 0.00 | 0.006 | 1.0000 |  |
|  | *Clostridium_sensu_stricto_1* | 0.02 | -0.01 | 0.012 | 0.0921 |  |
|  | *Dubosiella* | 0.11 | -0.28 | 0.144 | 0.0707 |  |
|  | *Enterococcus* | -0.03 | -0.45 | 0.480 | 0.9081 |  |
|  | *Erysipelotrichaceae_UCG-003* | -0.06^b^ | 0.00^a^ | 0.012 | 0.0010 |  |
|  | *Faecalibacterium* | 0.14 | 0.00 | 0.047 | 0.4591 |  |
|  | *Faecalibaculum* | -0.09^y^ | 0.01^z^ | 0.060 | 0.0496 |  |
|  | *Holdemanella* | -0.01 | 0.00 | 0.012 | 0.2338 |  |
|  | *Lachnoclostridium* | -0.06^y^ | 0.00^z^ | 0.012 | 0.0030 |  |
|  | Lachnospiraceae unclassified | 0.09^z^ | 0.00^y^ | 0.025 | 0.0263 |  |
|  | Lachnospiraceae uncultured | 0.03 | 0.00 | 0.015 | 0.4591 |  |
|  | *Lachnospiraceae_NK4A136_group* | 0.04^z^ | 0.00^y^ | 0.005 | <0.0001 |  |
|  | *Lactobacillus* | -0.27 | -1.63 | 1.068 | 0.1842 |  |
|  | *Megamonas* | 0.03^z^ | 0.00^y^ | 0.005 | <0.0001 |  |
|  | *Oribacterium* | 0.01 | 0.00 | 0.009 | 0.1377 |  |
|  | *Peptoclostridium* | 0.24 | 0.00 | 0.180 | 1.0000 |  |
|  | *Peptococcus* | 0.29 | 0.00 | 0.115 | 0.9516 |  |
|  | *Peptostreptococcus* | -0.11 | 0.00 | 0.106 | 0.1387 |  |
|  | *Phascolarctobacterium* | 0.02 | 0.00 | 0.018 | 0.1387 |  |
|  | *Romboutsia* | 0.01 | 0.00 | 0.022 | 0.4591 |  |
|  | Ruminococcaceae unclassified | 0.07^z^ | 0.00^y^ | 0.013 | 0.0031 |  |
|  | *Sellimonas* | 0.00 | 0.00 | 0.011 | 1.0000 |  |
|  | *Streptococcus* | -0.05 | 0.10 | 0.417 | 0.9162 |  |
|  | *Terrisporobacter* | 0.00^z^ | -0.09^y^ | 0.013 | 0.0002 |  |
|  | *Turicibacter* | -1.09^y^ | 0.03^z^ | 0.145 | 0.0005 |  |
| Fusobacteriota | | | 1.21^z^ | -0.02^y^ | 0.198 | 0.0048 |
|  | | *Cetobacterium* | -0.07 | 0.00 | 0.034 | 0.1387 |
|  | | *Fusobacterium* | 1.27^z^ | -0.02^y^ | 0.186 | 0.0048 |
| Proteobacteria | | | 0.09^y^ | 1.83^z^ | 0.302 | 0.0056 |
|  | | *Anaerobiospirillum* | 0.03 | 0.04 | 0.011 | 0.3870 |
|  | | *Escherichia-Shigella* | 0.00^y^ | 1.06^z^ | 0.195 | 0.0056 |
|  | | *Parasutterella* | 0.02^b^ | 0.63^a^ | 0.131 | 0.0034 |
|  | | *Proteus* | 0.00^y^ | 0.02^z^ | 0.007 | 0.0263 |
|  | | *Sutterella* | 0.04 | 0.08 | 0.020 | 0.1681 |

^1^ABX- = samples collected from dogs prior to antibiotic administration; ABX+ = samples collected after receiving metronidazole (20 mg/kg body weight twice daily) for two wk.
^2^SEM = pooled standard errors of the means.
^ab^Mean values within a row with unlike superscript letters differ using parametric analysis (P<0.05).
^yz^Mean values within a row with unlike superscript letters differ using non-parametric analysis (P<0.05).

# **Supplemental Table 3.** Change from baseline (0 hr) pH and SCFA concentrations (μmole/g, OMB) of tubes containing cellulose, pectin, beet pulp, or chicory pulp

|  |  | Δ0 to 6 hr | | Δ0 to 12 hr | | Δ0 to 18 hr | |  | p-value | | |
| --- | --- | --- | --- | --- | --- | --- | --- | --- | --- | --- | --- |
| Fiber |  | ABX-^1^ | ABX+ | ABX- | ABX+ | ABX- | ABX+ | SEM^2^ | Ant | Time | Ant*Time |
| Pectin | pH | -1.02^b^ | -0.88^a^ | -1.56^c^ | -0.80^a^ | -1.69^d^ | -1.02^b^ | 0.021 | <0.0001 | <0.0001 | <0.0001 |
|  | Acetate | 1233.46^b^ | 494.86^d^ | 2918.04^a^ | 485.78^d^ | 2935.25^a^ | 909.42^c^ | 58.582 | <0.0001 | <0.0001 | <0.0001 |
|  | Propionate | 72.67^c^ | -15.94^d^ | 769.63^b^ | 77.18^c^ | 894.03^a^ | 18.24^cd^ | 14.728 | <0.0001 | <0.0001 | <0.0001 |
|  | Butyrate | 46.15 | 5.40 | 243.48 | -117.46 | 581.09 | -402.45 | 17.864 | 0.0003 | 1.0000 | NA^3^ |
| Beet  pulp | pH | -0.38 | -0.26 | -0.56 | -0.41 | -0.73 | -0.65 | 0.021 | <0.0001 | <0.0001 | 0.0528 |
|  | Acetate | 1246.09^c^ | 300.50^e^ | 1698.44^b^ | 947.62^d^ | 1848.21^ab^ | 1886.16^a^ | 37.220 | <0.0001 | <0.0001 | <0.0001 |
|  | Propionate | 63.91^d^ | -4.74^d^ | 603.60^b^ | 27.21^d^ | 747.79^a^ | 473.54^c^ | 17.682 | <0.0001 | <0.0001 | <0.0001 |
|  | Butyrate | 91.07^c^ | 4.81^d^ | 624.90^b^ | -84.67^e^ | 743.47^a^ | -50.84^e^ | 8.059 | <0.0001 | <0.0001 | <0.0001 |
| Chicory pulp | pH | -0.09^a^ | -0.18^b^ | -0.18^b^ | -0.22^b^ | -0.33^c^ | -0.17^b^ | 0.014 | 0.3064 | <0.0001 | <0.0001 |
|  | Acetate | 246.26 | 147.07 | 581.65 | 465.94 | 977.07 | 732.06 | 31.442 | <0.0001 | <0.0001 | 0.0542 |
|  | Propionate | 71.91^d^ | -1.97^e^ | 320.22^b^ | 50.45^de^ | 487.16^a^ | 170.65^c^ | 13.934 | <0.0001 | <0.0001 | <0.0001 |
|  | Butyrate | 114.28^b^ | 10.71^c^ | 455.98^a^ | -3.19^c^ | 496.56^a^ | -173.98^d^ | 11.144 | <0.0001 | <0.0001 | <0.0001 |
| Cellulose | pH | 0.06 | 0.06 | 0.04 | 0.09 | 0.00 | 0.06 | 0.017 | 0.0554 | 0.3157 | NA |
|  | Acetate | -65.55^b^ | 23.83^ab^ | -54.02^b^ | 53.63^ab^ | 150.28^a^ | -9.94^b^ | 60.914 | 0.7434 | 0.1523 | 0.0231 |
|  | Propionate | -25.36^b^ | -7.29^ab^ | -17.07^ab^ | 7.94^ab^ | 36.51^a^ | -8.46^ab^ | 17.417 | 0.9458 | 0.0585 | 0.0188 |
|  | Butyrate | -20.40 | -2.91 | 13.07 | -8.80 | 21.59 | -43.77 | 38.152 | 0.3183 | 0.8495 | 0.3499 |

^1^ABX- = samples collected from dogs prior to antibiotic administration; ABX+ = samples collected after receiving metronidazole (20 mg/kg body weight twice daily) for two wk.
^2^SEM = pooled standard errors of the means.
^3^NA: data not normal, requiring non-parametric statistical analysis.
^a-e^Mean values within a row with unlike superscript letters differ using parametric analysis (P<0.05).

# **Supplemental Table 4.** Alpha diversity measures in tubes containing cellulose, pectin, beet pulp, or chicory pulp

|  |  | 0 hr | | 6 hr | | 12 hr | | 18 hr | |  | p-value | | |
| --- | --- | --- | --- | --- | --- | --- | --- | --- | --- | --- | --- | --- | --- |
| Fiber |  | ABX-^1^ | ABX+ | ABX- | ABX+ | ABX- | ABX+ | ABX- | ABX+ | SEM^2^ | Ant | Time | Ant*Time |
| Pectin | Shannon Diversity | 5.10^a^ | 3.99^c^ | 4.92^a^ | 3.60^d^ | 4.81^ab^ | 3.74^cd^ | 4.55^b^ | 4.03^c^ | 0.060 | <0.0001 | 0.0005 | <0.0001 |
|  | Faith's PD | 7.44^a^ | 4.12^c^ | 7.04^ab^ | 3.16^d^ | 6.72^ab^ | 4.43^c^ | 6.53^b^ | 4.96^c^ | 0.180 | <0.0001 | 0.0056 | <0.0001 |
|  | Pielou Evenness | 0.77 | 0.74 | 0.75 | 0.70 | 0.74 | 0.68 | 0.71 | 0.68 | 0.006 | <0.0001 | <0.0001 | 0.1928 |
| Beet pulp | Shannon Diversity | 5.38^a^ | 3.91^e^ | 5.20^b^ | 3.99^e^ | 5.23^ab^ | 4.38^d^ | 5.27^ab^ | 4.95^c^ | 0.032 | <0.0001 | <0.0001 | <0.0001 |
|  | Faith's PD | 7.69^a^ | 3.46^c^ | 7.35^a^ | 3.86^c^ | 7.26^a^ | 5.50^b^ | 7.51^a^ | 6.11^b^ | 0.172 | <0.0001 | <0.0001 | <0.0001 |
|  | Pielou Evenness | 0.80^a^ | 0.74^b^ | 0.78^a^ | 0.74^b^ | 0.79^a^ | 0.74^b^ | 0.78^a^ | 0.78^a^ | 0.007 | <0.0001 | 0.0375 | 0.0043 |
| Chicory pulp | Shannon Diversity | 5.37^a^ | 3.99^c^ | 5.25^a^ | 3.81^c^ | 5.38^a^ | 4.56^b^ | 5.33^a^ | 5.18^a^ | 0.068 | <0.0001 | <0.0001 | <0.0001 |
|  | Faith's PD | 7.78^a^ | 3.68^c^ | 7.31^a^ | 4.01^c^ | 7.84^a^ | 5.57^b^ | 7.71^a^ | 7.05^a^ | 0.261 | <0.0001 | <0.0001 | <0.0001 |
|  | Pielou Evenness | 0.80^a^ | 0.75^b^ | 0.79^ab^ | 0.70^c^ | 0.79^a^ | 0.75^b^ | 0.79^a^ | 0.78^ab^ | 0.009 | <0.0001 | 0.0010 | 0.0057 |
| Cellulose | Shannon Diversity | 5.31^a^ | 3.84^c^ | 5.30^a^ | 4.01^c^ | 5.31^a^ | 4.74^b^ | 5.36^a^ | 5.17^a^ | 0.064 | <0.0001 | <0.0001 | <0.0001 |
|  | Faith's PD | 7.76^a^ | 3.13^e^ | 7.63^a^ | 4.00^d^ | 7.79^a^ | 5.67^c^ | 7.86^a^ | 6.80^b^ | 0.201 | <0.0001 | <0.0001 | <0.0001 |
|  | Pielou Evenness | 0.78^a^ | 0.73^c^ | 0.79^a^ | 0.74^bc^ | 0.78^ab^ | 0.77^abc^ | 0.79^a^ | 0.79^a^ | 0.009 | 0.0004 | 0.0265 | 0.0253 |

^1^ABX- = samples collected from dogs prior to antibiotic administration; ABX+ = samples collected after receiving metronidazole (20 mg/kg body weight twice daily) for two wk.
^2^SEM = pooled standard errors of the means.
^a-e^Mean values within a row with unlike superscript letters differ using parametric analysis (P<0.05).

# **Supplemental Table 5.** Change from baseline (0 hr) bacterial phyla and genera relative abundances (% of sequences) of tubes containing pectin

|  |  | Δ0 to 6 hr | | Δ0 to 12 hr | | Δ0 to 18 hr | |  | p-value | | |
| --- | --- | --- | --- | --- | --- | --- | --- | --- | --- | --- | --- |
| Phyla | Genera | ABX-^1^ | ABX+ | ABX- | ABX+ | ABX- | ABX+ | SEM^2^ | Ant | Time | Ant*Time |
| Actinobacteridota | | 3.13^bc^ | 5.81^b^ | 2.70^bc^ | 11.27^a^ | 1.89^c^ | 10.55^a^ | 0.660 | <0.0001 | 0.0072 | 0.0009 |
|  | *Adlercreutzia* | -0.09 | 0.00 | -0.06 | 0.00 | -0.05 | 0.00 | 0.015 | 0.0002 | 0.3073 | 0.3073 |
|  | *Bifidobacterium* | 1.69^c^ | 7.61^b^ | 1.36^c^ | 6.87^b^ | 2.25^c^ | 13.65^a^ | 0.644 | <0.0001 | 0.0001 | 0.0010 |
|  | *Collinsella* | 0.62^a^ | -0.88^d^ | 0.52^ab^ | 0.00^c^ | 0.63^a^ | 0.39^b^ | 0.046 | <0.0001 | <0.0001 | <0.0001 |
|  | *Coriobacteriaceae_UCG-002* | 0.27 | 0.00 | 0.19 | 0.00 | 0.15 | 0.01 | 0.022 | 0.0002 | 0.8644 | NA^3^ |
|  | *Parvibacter* | 0.11 | 0.00 | 0.09 | 0.00 | 0.05 | 0.00 | 0.013 | 0.0001 | 0.7505 | NA |
|  | *Slackia* | -0.01 | -0.01 | -0.01 | -0.01 | 0.00 | 0.00 | 0.007 | 0.8064 | 0.2343 | 0.9652 |
| Bacteroidota | | 13.82^b^ | -0.10^c^ | 20.71^a^ | -5.19^d^ | 20.38^a^ | -6.29^d^ | 0.775 | <0.0001 | 0.3029 | <0.0001 |
|  | *Alloprevotella* | -0.06^a^ | -0.09^ab^ | -0.14^c^ | -0.13^bc^ | -0.23^d^ | -0.17^c^ | 0.009 | 0.0583 | <0.0001 | 0.0011 |
|  | *Bacteroides* | 0.80^a^ | 0.13^a^ | 0.36^a^ | -1.62^b^ | 1.32^a^ | -6.96^c^ | 0.316 | <0.0001 | <0.0001 | <0.0001 |
|  | *Muribaculaceae* | -0.79 | 0.00 | -1.48 | -0.03 | -1.12 | -0.80 | 0.060 | 0.0009 | 0.1392 | NA |
|  | *Parabacteroides* | -0.11^b^ | 0.00^a^ | -0.37^d^ | -0.02^a^ | -0.48^e^ | -0.16^c^ | 0.007 | <0.0001 | <0.0001 | <0.0001 |
|  | *Prevotella* | 21.91 | 0.15 | 22.88 | 1.55 | 13.36 | -2.88 | 0.797 | 0.0003 | 0.2277 | NA |
|  | *Prevotellaceae_Ga6A1_group* | -0.16 | 0.00 | -0.13 | 0.00 | -0.07 | -0.03 | 0.007 | 0.0003 | 0.9280 | NA |
| Firmicutes | | 5.91^cd^ | 13.30^bc^ | 2.12^d^ | 18.86^ab^ | 3.06^d^ | 27.41^a^ | 1.813 | <0.0001 | 0.0194 | 0.0019 |
|  | *[Eubacterium]_brachy_group* | -0.95 | 0.00 | -1.45 | -0.29 | -1.55 | -0.78 | 0.017 | 0.0003 | 0.1468 | NA |
|  | *[Ruminococcus]_gauvreauii_group* | -0.03^a^ | 0.00^a^ | -0.11^b^ | -0.11^b^ | 0.02^a^ | -0.22^c^ | 0.012 | <0.0001 | <0.0001 | <0.0001 |
|  | *[Ruminococcus]_gnavus_group* | 0.01^ab^ | 0.03^ab^ | 0.07^ab^ | -0.14^bc^ | 0.23^a^ | -0.41^c^ | 0.066 | 0.0004 | 0.2669 | 0.0014 |
|  | *[Ruminococcus]_torques_group* | 0.23^b^ | 0.00^c^ | 0.03^bc^ | -1.07^e^ | 0.84^a^ | -0.82^d^ | 0.055 | <0.0001 | <0.0001 | <0.0001 |
|  | *Allobaculum* | -3.18^bc^ | -2.14^b^ | -4.98^d^ | -0.98^a^ | -3.43^c^ | -0.45^a^ | 0.237 | <0.0001 | 0.0026 | 0.0001 |
|  | *Anaeroplasma* | 0.00 | -0.05 | 0.00 | 0.07 | 0.01 | -0.05 | 0.024 | 0.5655 | 0.0442 | 0.0559 |
|  | *Blautia* | 0.66^a^ | 0.06^a^ | -1.91^b^ | -1.94^b^ | 0.65^a^ | -3.26^c^ | 0.240 | <0.0001 | <0.0001 | <0.0001 |
|  | *Butyricicoccus* | -0.03^a^ | 0.00^a^ | 0.01^a^ | -0.51^c^ | 0.03^a^ | -0.34^b^ | 0.015 | <0.0001 | <0.0001 | <0.0001 |
|  | *Clostridium_sensu_stricto_1* | -0.38^b^ | 0.01^a^ | -0.62^c^ | -0.79^d^ | -1.47^e^ | -0.64^c^ | 0.013 | <0.0001 | <0.0001 | <0.0001 |
|  | *Dubosiella* | 1.26^ab^ | -0.75^e^ | 0.82^bc^ | -0.10^de^ | 1.92^a^ | 0.42^cd^ | 0.164 | <0.0001 | 0.0004 | 0.0223 |
|  | *Enterococcus* | 0.60 | 3.12 | 0.96 | 12.41 | 0.79 | 17.42 | 0.927 | 0.0054 | 0.1443 | NA |
|  | *Erysipelotrichaceae_UCG-003* | 0.09 | -0.03 | 0.11 | -0.05 | 0.12 | 0.01 | 0.011 | <0.0001 | 0.0128 | 0.1257 |
|  | *Faecalibacterium* | 3.49 | 0.06 | 3.09 | -0.45 | 3.14 | -4.20 | 0.332 | 0.0003 | 0.4579 | NA |
|  | *Faecalibaculum* | 0.42 | -0.09 | 0.36 | -0.03 | 0.67 | -0.02 | 0.106 | <0.0001 | 0.2528 | 0.4584 |
|  | *Holdemanella* | 0.11 | 0.00 | 0.21 | 0.01 | 0.14 | 0.00 | 0.024 | <0.0001 | 0.0954 | 0.1685 |
|  | *Lachnoclostridium* | -0.05 | 0.00 | -0.11 | 0.00 | 0.00 | -0.05 | 0.028 | 0.0101 | 0.5852 | NA |
|  | Lachnospiraceae unclassified | -0.64 | 0.00 | -1.12 | -0.83 | -0.88 | -0.63 | 0.016 | 0.0115 | 0.0080 | NA |
|  | Lachnospiraceae uncultured | 0.08 | 0.00 | -0.01 | -0.13 | -0.08 | -0.15 | 0.033 | 0.0832 | 0.0045 | NA |
|  | *Lachnospiraceae_NK4A136_group* | 0.00 | 0.00 | -0.03 | -0.02 | 0.01 | 0.00 | 0.008 | 0.5524 | 0.0076 | 0.2729 |
|  | *Lactobacillus* | -0.45^bc^ | -4.31^c^ | 0.40^bc^ | 2.91^b^ | 0.21^bc^ | 13.03^a^ | 1.052 | 0.0008 | <0.0001 | <0.0001 |
|  | *Megamonas* | -0.06 | 0.00 | -0.06 | 0.00 | -0.09 | 0.00 | 0.005 | 0.0001 | 0.6548 | NA |
|  | *Oribacterium* | -0.18 | 0.00 | -0.68 | -0.06 | -0.60 | -0.22 | 0.005 | 0.0050 | 0.0343 | NA |
|  | *Peptoclostridium* | -6.41^d^ | 0.01^a^ | -4.08^c^ | -5.39^d^ | -4.03^c^ | -1.94^b^ | 0.239 | <0.0001 | <0.0001 | <0.0001 |
|  | *Peptococcus* | -0.11 | 0.00 | -0.04 | 0.00 | -0.15 | -0.15 | 0.002 | 0.1361 | 0.0023 | NA |
|  | *Peptostreptococcus* | -2.00 | 0.00 | -2.02 | -1.94 | -1.72 | -4.62 | 0.072 | 0.8942 | 0.2331 | NA |
|  | *Phascolarctobacterium* | 0.29^a^ | 0.00^b^ | 0.26^a^ | 0.01^b^ | -0.04^b^ | -0.52^c^ | 0.040 | <0.0001 | <0.0001 | 0.0206 |
|  | *Romboutsia* | -0.46 | 0.00 | -0.42 | 0.00 | -0.41 | 0.00 | 0.009 | 0.0001 | 0.7259 | NA |
|  | Ruminococcaceae unclassified | 0.18^ab^ | 0.00^bc^ | 0.04^bc^ | -0.13^c^ | 0.35^a^ | -0.29^d^ | 0.053 | <0.0001 | 0.0200 | 0.0001 |
|  | *Sellimonas* | -0.12 | 0.00 | -0.16 | -0.14 | -0.12 | -0.17 | 0.011 | 0.8940 | 0.0378 | NA |
|  | *Streptococcus* | 10.35^c^ | 16.55^ab^ | 15.43^b^ | 16.18^b^ | 12.87^bc^ | 21.01^a^ | 0.825 | <0.0001 | 0.0034 | 0.0020 |
|  | *Terrisporobacter* | 0.00^b^ | -0.14^c^ | 0.00^b^ | 0.01^ab^ | 0.00^b^ | 0.06^a^ | 0.012 | 0.0326 | <0.0001 | <0.0001 |
|  | *Turicibacter* | -1.06^d^ | -0.08^a^ | -0.40^b^ | -0.06^a^ | -0.80^c^ | -0.03^a^ | 0.054 | <0.0001 | <0.0001 | 0.0001 |
| Fusobacteriota |  | -20.29^c^ | -0.03^a^ | -22.33^d^ | -8.44^b^ | -21.36^cd^ | -24.77^e^ | 0.269 | <0.0001 | <0.0001 | <0.0001 |
|  | *Cetobacterium* | -0.12^c^ | 0.00^b^ | 0.01^b^ | 0.00^b^ | 0.15^a^ | 0.00^b^ | 0.013 | 0.1054 | <0.0001 | <0.0001 |
|  | *Fusobacterium* | -22.21^d^ | 0.03^a^ | -23.87^e^ | -7.85^b^ | -17.91^c^ | -25.43^f^ | 0.272 | <0.0001 | <0.0001 | <0.0001 |
| Proteobacteria |  | -2.57 | -18.98 | -3.20 | -16.50 | -3.96 | -6.90 | 0.829 | 0.0003 | 1.0000 | NA |
|  | *Anaerobiospirillum* | -0.03 | -0.05 | -0.06 | -0.05 | 0.04 | -0.06 | 0.024 | 0.1023 | 0.4843 | NA |
|  | *Escherichia-Shigella* | -0.08 | -14.43 | -0.14 | -9.65 | -0.10 | -1.33 | 0.562 | 0.0003 | 0.6525 | NA |
|  | *Parasutterella* | -1.13 | -3.57 | -0.83 | -3.04 | -0.69 | -3.18 | 0.201 | 0.0003 | 0.4843 | NA |
|  | *Proteus* | 0.00 | -0.53 | 0.00 | -0.97 | 0.00 | -0.56 | 0.024 | 0.0001 | 0.6567 | NA |
|  | *Sutterella* | -1.79^b^ | -0.75^a^ | -2.41^c^ | -0.57^a^ | -2.53^c^ | -3.39^d^ | 0.080 | <0.0001 | <0.0001 | <0.0001 |

^1^ABX- = samples collected from dogs prior to antibiotic administration; ABX+ = samples collected after receiving metronidazole (20 mg/kg body weight twice daily) for two wk.
^2^SEM = pooled standard errors of the means.
^3^NA: data not normal, requiring non-parametric statistical analysis.
^a-f^Mean values within a row with unlike superscript letters differ using parametric analysis (P<0.05).

# **Supplemental Table 6.** Change from baseline (0 hr) bacterial phyla and genera relative abundances (% of sequences) of tubes containing beet pulp

|  |  | Δ0 to 6 hr | | Δ0 to 12 hr | | Δ0 to 18 hr | |  | p-value | | |
| --- | --- | --- | --- | --- | --- | --- | --- | --- | --- | --- | --- |
| Phyla | Genera | ABX-^1^ | ABX+ | ABX- | ABX+ | ABX- | ABX+ | SEM^2^ | Ant | Time | Ant*Time |
| Actinobacteridota | | 2.77^d^ | 6.83^c^ | 2.24^d^ | 18.73^a^ | 2.53^d^ | 11.17^b^ | 0.494 | <0.0001 | <0.0001 | <0.0001 |
|  | *Adlercreutzia* | -0.09 | 0.00 | -0.04 | 0.00 | -0.02 | 0.00 | 0.011 | 0.0029 | 0.3481 | NA^3^ |
|  | *Bifidobacterium* | 2.88^d^ | 7.75^c^ | 2.41^d^ | 18.82^a^ | 2.67^d^ | 11.18^b^ | 0.455 | <0.0001 | <0.0001 | <0.0001 |
|  | *Collinsella* | -0.04^a^ | -0.90^b^ | -0.15^a^ | -0.09^a^ | -0.16^a^ | -0.01^a^ | 0.060 | 0.0007 | <0.0001 | <0.0001 |
|  | *Coriobacteriaceae_UCG-002* | -0.05 | 0.00 | 0.00 | 0.00 | 0.04 | 0.00 | 0.016 | 0.6712 | 0.1512 | NA |
|  | *Parvibacter* | 0.06 | 0.00 | 0.06 | 0.00 | 0.02 | 0.00 | 0.018 | 0.0338 | 0.5708 | NA |
|  | *Slackia* | -0.06^b^ | 0.00^a^ | -0.03^ab^ | -0.02^ab^ | 0.01^a^ | 0.00^a^ | 0.010 | 0.0353 | 0.0152 | 0.0217 |
| Bacteroidota | | 15.01^a^ | 0.07^c^ | 13.13^ab^ | 2.55^c^ | 10.86^b^ | 10.98^b^ | 0.667 | <0.0001 | 0.0004 | <0.0001 |
|  | *Alloprevotella* | -0.12 | 0.03 | -0.19 | -0.02 | -0.22 | -0.12 | 0.017 | <0.0001 | <0.0001 | 0.2052 |
|  | *Bacteroides* | 3.11^a^ | 0.01^b^ | 4.03^a^ | 4.18^a^ | 4.34^a^ | -3.13^c^ | 0.626 | <0.0001 | 0.0002 | 0.0001 |
|  | *Muribaculaceae* | -0.58^b^ | 0.01^a^ | -1.26^c^ | 0.21^a^ | -1.43^c^ | -0.79^b^ | 0.089 | <0.0001 | <0.0001 | 0.0005 |
|  | *Parabacteroides* | -0.01 | 0.00 | -0.20 | 0.05 | -0.43 | -0.16 | 0.021 | 0.0238 | 0.0177 | NA |
|  | *Prevotella* | 10.85^bc^ | 1.23^d^ | 8.47^c^ | 15.49^a^ | 13.37^ab^ | -2.87^e^ | 0.663 | <0.0001 | <0.0001 | <0.0001 |
|  | *Prevotellaceae_Ga6A1_group* | -0.10 | 0.00 | -0.08 | 0.00 | -0.01 | -0.03 | 0.006 | 0.0214 | 0.8162 | NA |
| Firmicutes | | 3.87^ab^ | 1.90^b^ | 8.30^a^ | -5.00^c^ | 9.10^a^ | 1.58^b^ | 1.289 | <0.0001 | 0.0407 | 0.0032 |
|  | *[Eubacterium]_brachy_group* | -0.97^d^ | 0.00^a^ | -1.45^e^ | -0.28^b^ | -1.55^e^ | -0.53^c^ | 0.025 | <0.0001 | <0.0001 | 0.0038 |
|  | *[Ruminococcus]_gauvreauii_group* | 0.04^a^ | 0.00^a^ | 0.10^a^ | 0.02^a^ | 0.06^a^ | -0.22^b^ | 0.033 | <0.0001 | 0.0008 | 0.0021 |
|  | *[Ruminococcus]_gnavus_group* | -0.16^ab^ | -0.01^a^ | -0.13^a^ | -0.08^a^ | -0.23^b^ | -0.40^b^ | 0.057 | 0.7662 | 0.0012 | 0.0189 |
|  | *[Ruminococcus]_torques_group* | 1.26^a^ | 0.23^b^ | 1.03^a^ | 0.92^a^ | 1.15^a^ | -0.82^c^ | 0.133 | <0.0001 | 0.0003 | 0.0001 |
|  | *Allobaculum* | -3.22^d^ | -0.96^b^ | -3.80^d^ | 0.10^a^ | -2.12^c^ | -0.08^a^ | 0.173 | <0.0001 | 0.0003 | 0.0003 |
|  | *Anaeroplasma* | 0.00 | 0.27 | -0.02 | 0.27 | -0.02 | 0.01 | 0.035 | 0.0087 | 0.0459 | NA |
|  | *Blautia* | 3.60^a^ | 0.02^b^ | 3.31^a^ | -2.10^d^ | 3.35^a^ | -0.57^c^ | 0.125 | <0.0001 | <0.0001 | <0.0001 |
|  | *Butyricicoccus* | -0.02^a^ | 0.00^a^ | 0.02^a^ | -0.53^c^ | 0.00^a^ | -0.11^b^ | 0.013 | <0.0001 | <0.0001 | <0.0001 |
|  | *Clostridium_sensu_stricto_1* | -0.25 | -0.03 | -0.52 | -0.15 | -1.41 | -0.17 | 0.143 | 0.0070 | 0.0945 | NA |
|  | *Dubosiella* | -0.35^d^ | 0.24^bc^ | -0.16^cd^ | 1.13^a^ | 0.10^bcd^ | 0.57^ab^ | 0.121 | <0.0001 | 0.0031 | 0.0122 |
|  | *Enterococcus* | 0.10 | -3.63 | 0.07 | -2.28 | -0.04 | -2.98 | 0.496 | <0.0001 | 0.3847 | 0.3621 |
|  | *Erysipelotrichaceae_UCG-003* | 0.05 | 0.10 | 0.08 | 0.03 | 0.05 | 0.04 | 0.017 | 0.9741 | 0.3452 | 0.0619 |
|  | *Faecalibacterium* | 7.36^b^ | 1.42^d^ | 7.06^b^ | 9.74^a^ | 4.92^c^ | -4.20^e^ | 0.276 | <0.0001 | <0.0001 | <0.0001 |
|  | *Faecalibaculum* | -0.06 | 0.11 | 0.01 | 0.12 | 0.07 | 0.17 | 0.067 | 0.0273 | 0.3226 | 0.7837 |
|  | *Holdemanella* | -0.04^b^ | 0.00^ab^ | 0.03^a^ | 0.02^ab^ | 0.05^a^ | 0.00^ab^ | 0.013 | 0.5556 | 0.0094 | 0.0177 |
|  | *Lachnoclostridium* | 0.29 | 0.00 | 0.26 | 0.15 | 0.08 | -0.05 | 0.062 | 0.0047 | 0.0303 | 0.3347 |
|  | Lachnospiraceae unclassified | 0.27^a^ | 0.00^bc^ | 0.11^ab^ | -0.62^d^ | -0.22^c^ | -0.63^d^ | 0.051 | <0.0001 | <0.0001 | 0.0014 |
|  | Lachnospiraceae uncultured | 0.09^ab^ | 0.00^bc^ | 0.02^bc^ | 0.21^a^ | -0.12^c^ | -0.15^d^ | 0.033 | 0.3274 | <0.0001 | 0.0024 |
|  | *Lachnospiraceae_NK4A136_group* | 0.11 | 0.00 | 0.09 | -0.02 | 0.03 | 0.00 | 0.014 | 0.0003 | 0.5124 | NA |
|  | *Lactobacillus* | -0.50^b^ | -10.74^d^ | 0.17^b^ | -7.40^c^ | 0.81^b^ | 15.63^a^ | 0.576 | 0.0392 | <0.0001 | <0.0001 |
|  | *Megamonas* | -0.03 | 0.00 | -0.03 | 0.00 | -0.05 | 0.00 | 0.005 | 0.0001 | 0.8458 | NA |
|  | *Oribacterium* | -0.15^b^ | 0.00^a^ | -0.57^d^ | 0.01^a^ | -0.60^d^ | -0.22^c^ | 0.014 | <0.0001 | <0.0001 | <0.0001 |
|  | *Peptoclostridium* | -8.01^d^ | 1.37^a^ | -5.62^c^ | -4.86^c^ | -4.98^c^ | -1.94^b^ | 0.197 | <0.0001 | <0.0001 | <0.0001 |
|  | *Peptococcus* | -0.06^b^ | 0.00^a^ | -0.01^a^ | 0.01^a^ | -0.12^c^ | -0.15^c^ | 0.010 | 0.1025 | <0.0001 | 0.0013 |
|  | *Peptostreptococcus* | -2.22^d^ | 0.69^a^ | -1.91^c^ | -0.69^b^ | -2.23^d^ | -4.61^e^ | 0.062 | <0.0001 | <0.0001 | <0.0001 |
|  | *Phascolarctobacterium* | 0.43^b^ | 0.02^c^ | 0.32^b^ | 1.33^a^ | -0.03^c^ | -0.52^d^ | 0.049 | 0.3284 | <0.0001 | <0.0001 |
|  | *Romboutsia* | -0.26 | 0.00 | -0.19 | 0.00 | -0.19 | 0.00 | 0.018 | 0.0001 | 0.7068 | NA |
|  | Ruminococcaceae unclassified | 0.39^a^ | 0.02^b^ | 0.46^a^ | 0.01^b^ | 0.38^a^ | -0.29^c^ | 0.054 | <0.0001 | 0.0007 | 0.0005 |
|  | *Sellimonas* | -0.10 | 0.00 | -0.17 | -0.09 | -0.14 | -0.17 | 0.014 | 0.1693 | 0.0127 | NA |
|  | *Streptococcus* | 8.71^ab^ | 4.51^bc^ | 10.88^a^ | 1.01^c^ | 9.60^a^ | 12.00^a^ | 0.906 | 0.0002 | 0.0003 | <0.0001 |
|  | *Terrisporobacter* | 0.00 | -0.05 | 0.00 | 0.03 | 0.00 | 0.21 | 0.012 | 0.2026 | 0.0226 | NA |
|  | *Turicibacter* | 0.20^c^ | -0.03^cd^ | 0.98^a^ | -0.06^d^ | 0.59^b^ | -0.01^cd^ | 0.049 | <0.0001 | <0.0001 | <0.0001 |
| Fusobacteriota |  | -18.99^d^ | 0.04^a^ | -20.41^d^ | -5.40^b^ | -18.56^d^ | -15.17^c^ | 0.687 | <0.0001 | <0.0001 | <0.0001 |
|  | *Cetobacterium* | -0.15 | 0.00 | -0.04 | 0.00 | 0.02 | 0.00 | 0.015 | 0.0338 | 0.1143 | NA |
|  | *Fusobacterium* | -20.18^c^ | 3.07^a^ | -20.98^c^ | 1.74^a^ | -16.55^b^ | -25.35^d^ | 0.685 | <0.0001 | <0.0001 | <0.0001 |
| Proteobacteria |  | -2.66 | -8.85 | -3.26 | -10.88 | -3.93 | -8.55 | 0.435 | 0.0003 | 0.5045 | NA |
|  | *Anaerobiospirillum* | -0.06 | -0.04 | -0.07 | 0.13 | -0.01 | 0.00 | 0.022 | 0.0851 | 0.4758 | NA |
|  | *Escherichia-Shigella* | -0.07 | -8.41 | -0.09 | -8.22 | -0.11 | -3.29 | 0.232 | 0.0003 | 0.9322 | NA |
|  | *Parasutterella* | -1.55^b^ | -1.60^b^ | -1.38^b^ | -2.90^b^ | -0.99^a^ | -0.37^a^ | 0.169 | 0.0406 | <0.0001 | 0.0001 |
|  | *Proteus* | 0.00 | -0.14 | 0.00 | -0.86 | 0.00 | -0.19 | 0.020 | 0.0001 | 0.6394 | NA |
|  | *Sutterella* | -1.42^b^ | 0.16^a^ | -1.84^bc^ | -0.28^a^ | -2.26^c^ | -2.07^c^ | 0.094 | <0.0001 | <0.0001 | <0.0001 |

^1^ABX- = samples collected from dogs prior to antibiotic administration; ABX+ = samples collected after receiving metronidazole (20 mg/kg body weight twice daily) for two wk.
^2^SEM = pooled standard errors of the means.
^3^NA: data not normal, requiring non-parametric statistical analysis.
^a-e^Mean values within a row with unlike superscript letters differ using parametric analysis (P<0.05).

# **Supplemental Table 7.** Change from baseline (0 hr) bacterial phyla and genera relative abundances (% of sequences) of tubes containing chicory pulp

|  |  | Δ0 to 6 hr | | Δ0 to 12 hr | | Δ0 to 18 hr | |  | p-value | | |
| --- | --- | --- | --- | --- | --- | --- | --- | --- | --- | --- | --- |
| Phyla | Genera | ABX-^1^ | ABX+ | ABX- | ABX+ | ABX- | ABX+ | SEM^2^ | Ant | Time | Ant*Time |
| Actinobacteridota | | 0.67 | -3.01 | 0.16 | -1.46 | -0.08 | -1.77 | 0.720 | 0.0003 | 0.8948 | NA^3^ |
|  | *Adlercreutzia* | -0.06 | 0.00 | 0.01 | 0.00 | -0.01 | 0.00 | 0.011 | 0.6712 | 0.1315 | NA |
|  | *Bifidobacterium* | 0.54 | -2.23 | 0.14 | -1.37 | -0.06 | -1.63 | 0.634 | 0.0003 | 0.6119 | NA |
|  | *Collinsella* | 0.16^a^ | -0.74^b^ | 0.06^a^ | -0.12^a^ | 0.03^a^ | -0.13^a^ | 0.106 | 0.0004 | 0.0518 | 0.0063 |
|  | *Coriobacteriaceae_UCG-002* | 0.03 | 0.00 | -0.04 | 0.00 | -0.01 | 0.00 | 0.029 | 0.6712 | 0.0829 | NA |
|  | *Parvibacter* | 0.01 | 0.00 | 0.00 | 0.01 | -0.03 | 0.00 | 0.017 | 0.5589 | 0.4384 | 0.5123 |
|  | *Slackia* | -0.02^ab^ | 0.03^a^ | 0.00^ab^ | -0.03^b^ | -0.01^ab^ | -0.02^ab^ | 0.012 | 0.5570 | 0.2184 | 0.0207 |
| Bacteroidota | | 4.93^a^ | 0.25^bc^ | 6.66^a^ | -1.69^c^ | 7.04^a^ | 4.08^ab^ | 0.864 | <0.0001 | 0.0058 | 0.0254 |
|  | *Alloprevotella* | -0.02 | 0.22 | -0.08 | 0.10 | -0.13 | -0.01 | 0.024 | <0.0001 | <0.0001 | 0.0874 |
|  | *Bacteroides* | -0.12^ab^ | 0.02^ab^ | 1.38^a^ | -0.86^b^ | 1.36^a^ | -0.05^ab^ | 0.349 | 0.0015 | 0.1725 | 0.0155 |
|  | *Muribaculaceae* | -0.58 | 0.00 | -0.72 | -0.01 | -0.73 | -0.44 | 0.083 | <0.0001 | 0.0115 | 0.0792 |
|  | *Parabacteroides* | -0.01^b^ | 0.00^b^ | -0.16^c^ | 0.20^a^ | -0.44^d^ | -0.16^c^ | 0.018 | <0.0001 | <0.0001 | <0.0001 |
|  | *Prevotella* | 6.32^a^ | 1.92^b^ | 7.04^a^ | 4.98^ab^ | 6.15^a^ | -2.88^c^ | 0.686 | <0.0001 | 0.0001 | 0.0009 |
|  | *Prevotellaceae_Ga6A1_group* | -0.12 | 0.00 | -0.11 | 0.00 | -0.03 | -0.03 | 0.010 | 0.0044 | 0.8361 | NA |
| Firmicutes | | 10.62 | 8.65 | 8.70 | 12.27 | 8.84 | 10.48 | 1.954 | 0.5114 | 0.8858 | 0.3848 |
|  | *[Eubacterium]_brachy_group* | -0.52^a^ | 0.00^b^ | -1.04^d^ | -0.17^c^ | -1.09^d^ | -0.06^bc^ | 0.027 | <0.0001 | <0.0001 | <0.0001 |
|  | *[Ruminococcus]_gauvreauii_group* | 0.21^ab^ | 0.09^bc^ | 0.28^a^ | 0.04^c^ | 0.26^a^ | -0.22^d^ | 0.032 | <0.0001 | 0.0009 | 0.0002 |
|  | *[Ruminococcus]_gnavus_group* | 0.16^ab^ | 0.22^ab^ | 0.30^ab^ | -0.10^bc^ | 0.43^a^ | -0.36^c^ | 0.095 | 0.0004 | 0.2914 | 0.0027 |
|  | *[Ruminococcus]_torques_group* | 0.30^ab^ | 0.66^a^ | 0.06^b^ | -0.41^c^ | 0.53^a^ | -0.82^d^ | 0.084 | <0.0001 | <0.0001 | <0.0001 |
|  | *Allobaculum* | -2.50^b^ | -1.09^a^ | -4.55^d^ | -0.73^a^ | -3.85^c^ | -0.56^a^ | 0.128 | <0.0001 | 0.0001 | <0.0001 |
|  | *Anaeroplasma* | -0.01^ab^ | 0.04^a^ | -0.01^ab^ | -0.07^b^ | 0.00^ab^ | -0.05^ab^ | 0.021 | 0.2330 | 0.0450 | 0.0267 |
|  | *Blautia* | 3.97 | 0.01 | 3.86 | -0.79 | 4.21 | -0.01 | 0.272 | <0.0001 | 0.0465 | 0.2886 |
|  | *Butyricicoccus* | 0.02^a^ | 0.00^a^ | 0.03^a^ | -0.41^b^ | 0.07^a^ | -0.01^a^ | 0.032 | <0.0001 | <0.0001 | <0.0001 |
|  | *Clostridium_sensu_stricto_1* | -0.16^cd^ | 0.02^c^ | -0.46^d^ | 6.18^a^ | -1.13^e^ | 2.28^b^ | 0.277 | <0.0001 | <0.0001 | <0.0001 |
|  | *Dubosiella* | 0.64^a^ | -0.05^b^ | 0.70^a^ | 0.76^a^ | 0.65^a^ | 0.57^a^ | 0.141 | 0.0409 | 0.0156 | 0.0282 |
|  | *Enterococcus* | 1.68 | 10.95 | 1.29 | 8.38 | 2.06 | 7.24 | 1.572 | <0.0001 | 0.4698 | 0.2697 |
|  | *Erysipelotrichaceae_UCG-003* | 0.03^a^ | 0.03^a^ | 0.06^a^ | -0.04^b^ | 0.04^a^ | -0.02^b^ | 0.010 | <0.0001 | 0.0629 | 0.0009 |
|  | *Faecalibacterium* | 1.83^b^ | -0.01^c^ | 4.38^a^ | 0.32^c^ | 5.56^a^ | -0.60^c^ | 0.280 | <0.0001 | 0.0002 | <0.0001 |
|  | *Faecalibaculum* | 0.45^b^ | -0.04^c^ | 0.86^a^ | -0.04^c^ | 0.55^ab^ | 0.00^c^ | 0.079 | <0.0001 | 0.0432 | 0.0276 |
|  | *Holdemanella* | -0.01 | 0.00 | 0.01 | 0.00 | 0.00 | 0.00 | 0.012 | 0.2028 | 0.9802 | NA |
|  | *Lachnoclostridium* | 0.06 | 0.00 | -0.04 | 0.00 | 0.02 | -0.05 | 0.020 | 0.2234 | 0.0747 | NA |
|  | Lachnospiraceae unclassified | -0.29 | 0.00 | -0.24 | -0.41 | 0.04 | -0.09 | 0.097 | 0.9279 | 0.0262 | 0.0644 |
|  | Lachnospiraceae uncultured | 0.14^a^ | 0.00^ab^ | 0.06^ab^ | -0.11^b^ | -0.03^ab^ | 0.12^a^ | 0.051 | 0.1642 | 0.1347 | 0.0088 |
|  | *Lachnospiraceae_NK4A136_group* | 0.17 | 0.00 | 0.15 | -0.02 | 0.22 | 0.06 | 0.018 | 0.0003 | 0.3093 | NA |
|  | *Lactobacillus* | 1.15^a^ | -4.54^b^ | 0.82^a^ | 1.27^a^ | 0.41^a^ | 1.01^a^ | 0.481 | 0.0024 | 0.0003 | <0.0001 |
|  | *Megamonas* | -0.04 | 0.00 | -0.06 | 0.00 | -0.07 | 0.00 | 0.005 | 0.0001 | 0.7798 | NA |
|  | *Oribacterium* | -0.05^b^ | 0.01^b^ | -0.49^d^ | 0.15^a^ | -0.55^e^ | -0.22^c^ | 0.013 | <0.0001 | <0.0001 | <0.0001 |
|  | *Peptoclostridium* | -5.71^d^ | 3.31^a^ | -2.82^b^ | -4.03^c^ | -1.68^b^ | -1.93^b^ | 0.249 | <0.0001 | <0.0001 | <0.0001 |
|  | *Peptococcus* | -0.02^b^ | 0.00^b^ | -0.01^b^ | 0.09^a^ | -0.09^c^ | -0.15^d^ | 0.009 | 0.0145 | <0.0001 | <0.0001 |
|  | *Peptostreptococcus* | -1.74^b^ | 2.17^a^ | -1.57^b^ | 1.49^a^ | -1.79^b^ | -4.61^c^ | 0.155 | <0.0001 | <0.0001 | <0.0001 |
|  | *Phascolarctobacterium* | 0.16^bc^ | 0.02^c^ | 0.26^b^ | 0.99^a^ | 0.25^b^ | -0.52^d^ | 0.047 | 0.1226 | <0.0001 | <0.0001 |
|  | *Romboutsia* | -0.19 | 0.00 | -0.13 | 0.00 | -0.06 | 0.00 | 0.014 | 0.0001 | 0.5824 | NA |
|  | Ruminococcaceae unclassified | 0.25^a^ | 0.17^a^ | 0.40^a^ | 0.31^a^ | 0.20^a^ | -0.29^b^ | 0.062 | 0.0009 | 0.0001 | 0.0089 |
|  | *Sellimonas* | -0.06^b^ | 0.04^a^ | -0.08^b^ | 0.04^a^ | -0.09^b^ | -0.17^c^ | 0.016 | 0.0041 | <0.0001 | <0.0001 |
|  | *Streptococcus* | 5.84^c^ | 0.09^d^ | 7.44^ab^ | 0.32^d^ | 7.66^a^ | 6.40^bc^ | 0.231 | <0.0001 | <0.0001 | <0.0001 |
|  | *Terrisporobacter* | 0.00 | -0.20 | 0.00 | -0.07 | 0.00 | 0.12 | 0.013 | 0.2028 | 0.0227 | NA |
|  | *Turicibacter* | -0.76 | -0.07 | -0.09 | -0.09 | 0.25 | -0.01 | 0.084 | 0.5076 | 0.0267 | NA |
| Fusobacteriota |  | -13.93 | -0.02 | -13.55 | -2.54 | -12.81 | -8.74 | 0.561 | 0.0003 | 0.7500 | NA |
|  | *Cetobacterium* | -0.14 | 0.00 | 0.01 | 0.00 | 0.05 | 0.00 | 0.020 | 0.6712 | 0.0388 | NA |
|  | *Fusobacterium* | -13.79 | -0.02 | -13.53 | -2.54 | -12.89 | -8.74 | 0.558 | 0.0003 | 0.6215 | NA |
| Proteobacteria |  | -2.29 | -5.88 | -1.97 | -6.58 | -2.98 | -4.04 | 1.081 | 0.0023 | 0.7961 | NA |
|  | *Anaerobiospirillum* | -0.05^ab^ | -0.02^a^ | -0.12^b^ | 0.01^a^ | -0.03^a^ | 0.02^a^ | 0.020 | 0.0002 | 0.0039 | 0.0163 |
|  | *Escherichia-Shigella* | -0.04 | -6.51 | -0.04 | -5.73 | 0.08 | -1.60 | 0.859 | 0.0003 | 0.3235 | NA |
|  | *Morganella* | 0.00 | 0.01 | 0.00 | -0.07 | 0.00 | 0.01 | 0.013 | 0.6513 | 0.0817 | NA |
|  | *Parasutterella* | -1.17^b^ | -0.46^b^ | -0.98^b^ | -1.52^b^ | -0.90^b^ | 0.77^a^ | 0.242 | 0.0067 | 0.0011 | 0.0018 |
|  | *Proteus* | 0.00 | 0.01 | 0.00 | -0.72 | 0.00 | -0.12 | 0.031 | 0.0338 | 0.0961 | NA |
|  | *Sutterella* | -0.51^b^ | 0.72^a^ | -1.46^c^ | 0.99^a^ | -2.01^c^ | -2.23^c^ | 0.191 | <0.0001 | <0.0001 | 0.0001 |

^1^ABX- = samples collected from dogs prior to antibiotic administration; ABX+ = samples collected after receiving metronidazole (20 mg/kg body weight twice daily) for two wk.
^2^SEM = pooled standard errors of the means.
^3^NA: data not normal, requiring non-parametric statistical analysis.
^a-e^Mean values within a row with unlike superscript letters differ using parametric analysis (P<0.05).

# **Supplemental Table 8.** Change from baseline (0 hr) bacterial phyla and genera relative abundances (% of sequences) of tubes containing cellulose

|  |  | Δ0 to 6 hr | | Δ0 to 12 hr | | Δ0 to 18 hr | |  | p-value | | |
| --- | --- | --- | --- | --- | --- | --- | --- | --- | --- | --- | --- |
| Phyla | Genera | ABX-^1^ | ABX+ | ABX- | ABX+ | ABX- | ABX+ | SEM^2^ | Ant | Time | Ant*Time |
| Actinobacteridota | | 0.48^b^ | 2.68^a^ | -0.10^b^ | 0.74^b^ | -0.05^b^ | 0.58^b^ | 0.308 | <0.0001 | <0.0001 | 0.0033 |
|  | *Adlercreutzia* | -0.02 | 0.00 | 0.01 | 0.00 | 0.04 | 0.00 | 0.004 | 0.6712 | 0.0326 | NA^3^ |
|  | *Bifidobacterium* | -0.02^b^ | -3.22^c^ | 0.02^b^ | -3.14^c^ | -0.05^b^ | 9.02^a^ | 0.266 | 0.0004 | <0.0001 | <0.0001 |
|  | *Collinsella* | -0.26^bc^ | -0.58^c^ | -0.03^b^ | 0.03^b^ | 0.15^b^ | 1.87^a^ | 0.102 | <0.0001 | <0.0001 | <0.0001 |
|  | *Coriobacteriaceae_UCG-002* | 0.05 | 0.00 | 0.06 | 0.00 | 0.16 | 0.00 | 0.027 | 0.0014 | 0.1020 | 0.1020 |
|  | *Parvibacter* | 0.04 | 0.00 | 0.03 | 0.01 | 0.04 | 0.00 | 0.016 | 0.0376 | 0.8784 | NA |
|  | *Slackia* | 0.01 | 0.00 | 0.05 | -0.01 | 0.08 | 0.03 | 0.013 | 0.0375 | 0.0210 | NA |
| Bacteroidota | | 0.99 | 0.12 | 0.65 | -1.27 | 0.30 | 0.78 | 0.686 | 0.1930 | 0.3742 | 0.2488 |
|  | *Alloprevotella* | -0.03^a^ | 0.06^a^ | 0.01^a^ | 0.09^a^ | -0.29^b^ | 0.01^a^ | 0.025 | <0.0001 | <0.0001 | 0.0016 |
|  | *Bacteroides* | 0.49^b^ | 1.98^b^ | 0.58^b^ | 4.96^a^ | 0.17^b^ | -6.91^c^ | 0.395 | 0.2374 | <0.0001 | <0.0001 |
|  | *Muribaculaceae* | 0.59 | 0.03 | 0.00 | 0.51 | -0.43 | -0.80 | 0.199 | 0.8249 | 0.0189 | NA |
|  | *Parabacteroides* | 0.25 | 0.00 | 0.03 | 0.26 | -0.33 | -0.16 | 0.028 | 0.8942 | 0.0026 | NA |
|  | *Prevotella* | -0.07^c^ | 2.03^a^ | 0.14^bc^ | 0.39^bc^ | 0.71^b^ | -2.89^d^ | 0.127 | 0.0015 | <0.0001 | <0.0001 |
|  | *Prevotellaceae_Ga6A1_group* | -0.02^b^ | 0.00^b^ | 0.01^ab^ | 0.04^ab^ | 0.08^a^ | -0.03^b^ | 0.015 | 0.1703 | 0.0896 | 0.0014 |
| Firmicutes | | 1.25 | -3.41 | 0.39 | 3.97 | -0.19 | -1.49 | 1.496 | 0.5292 | 0.0908 | 0.0516 |
|  | *[Eubacterium]_brachy_group* | 0.26 | 0.15 | 0.08 | 0.32 | -0.54 | -0.78 | 0.106 | 0.6855 | <0.0001 | 0.1010 |
|  | *[Ruminococcus]_gauvreauii_group* | -0.03^b^ | 0.13^a^ | -0.07^b^ | 0.14^a^ | 0.00^b^ | -0.22^c^ | 0.023 | 0.0258 | <0.0001 | <0.0001 |
|  | *[Ruminococcus]_gnavus_group* | -0.20^bc^ | 0.21^a^ | 0.02^ab^ | 0.03^ab^ | 0.01^ab^ | -0.29^c^ | 0.056 | 0.3651 | 0.0211 | 0.0002 |
|  | *[Ruminococcus]_torques_group* | 0.09^c^ | 0.87^a^ | -0.34^d^ | -0.18^cd^ | 0.48^b^ | -0.82^e^ | 0.063 | 0.0355 | <0.0001 | <0.0001 |
|  | *Allobaculum* | 0.12^a^ | 0.18^a^ | -0.63^b^ | 0.21^a^ | 0.39^a^ | 0.14^a^ | 0.084 | 0.0085 | 0.0003 | <0.0001 |
|  | *Anaeroplasma* | -0.01 | 0.04 | -0.01 | -0.06 | -0.01 | 0.08 | 0.030 | 0.6850 | 0.2645 | NA |
|  | *Blautia* | 0.33^b^ | 2.17^a^ | -0.42^c^ | 1.00^b^ | -0.53^c^ | -3.25^d^ | 0.150 | 0.1611 | <0.0001 | <0.0001 |
|  | *Butyricicoccus* | -0.04^b^ | 0.40^a^ | -0.01^b^ | -0.10^b^ | -0.01^b^ | -0.34^c^ | 0.032 | 0.8084 | <0.0001 | <0.0001 |
|  | *Clostridium_sensu_stricto_1* | 0.30^bc^ | 0.75^ab^ | 1.00^a^ | -0.05^cd^ | -1.07^e^ | -0.61^de^ | 0.143 | 0.7098 | <0.0001 | 0.0002 |
|  | *Dubosiella* | 0.13^bc^ | -0.11^c^ | 0.04^bc^ | 0.41^bc^ | 0.52^b^ | 2.12^a^ | 0.172 | 0.0003 | <0.0001 | 0.0001 |
|  | *Enterococcus* | -0.26^b^ | -6.27^c^ | 0.09^b^ | -7.75^c^ | -0.01^b^ | 9.76^a^ | 0.480 | 0.0017 | <0.0001 | <0.0001 |
|  | *Erysipelotrichaceae_UCG-003* | 0.07^a^ | -0.03^ab^ | 0.05^a^ | -0.28^c^ | 0.06^a^ | -0.06^bc^ | 0.025 | <0.0001 | 0.0003 | 0.0009 |
|  | *Faecalibacterium* | 0.30^c^ | 0.41^c^ | -0.94^d^ | 3.70^a^ | 1.42^b^ | -4.20^e^ | 0.279 | 0.1083 | <0.0001 | <0.0001 |
|  | *Faecalibaculum* | -0.06 | -0.08 | 0.01 | -0.02 | 0.38 | 0.06 | 0.090 | 0.0896 | 0.0124 | 0.1645 |
|  | *Holdemanella* | -0.08 | 0.00 | 0.00 | 0.00 | 0.04 | 0.00 | 0.011 | 0.2028 | 0.1289 | NA |
|  | *Lachnoclostridium* | 0.27 | 0.00 | 0.24 | 0.09 | 0.12 | -0.05 | 0.042 | 0.0002 | 0.0269 | 0.3483 |
|  | Lachnospiraceae unclassified | -0.17^b^ | 0.65^a^ | -0.52^c^ | -0.13^b^ | -0.66^c^ | -0.63^c^ | 0.064 | <0.0001 | <0.0001 | 0.0002 |
|  | Lachnospiraceae uncultured | -0.12 | 0.03 | -0.05 | 0.11 | -0.23 | -0.15 | 0.047 | 0.0064 | 0.0018 | 0.6797 |
|  | *Lachnospiraceae_NK4A136_group* | -0.07 | 0.00 | -0.04 | 0.00 | -0.02 | 0.00 | 0.013 | 0.0014 | 0.1638 | 0.1573 |
|  | *Lactobacillus* | -0.72^b^ | -8.22^c^ | 0.34^b^ | -8.34^c^ | 0.63^b^ | 17.50^a^ | 0.722 | 0.6988 | <0.0001 | <0.0001 |
|  | *Megamonas* | -0.02 | 0.00 | -0.01 | 0.00 | -0.03 | 0.00 | 0.005 | 0.0029 | 0.5880 | NA |
|  | *Oribacterium* | 0.33^a^ | 0.05^c^ | -0.09^d^ | 0.16^b^ | -0.38^f^ | -0.22^e^ | 0.023 | 0.0242 | <0.0001 | <0.0001 |
|  | *Peptoclostridium* | -2.67^c^ | 5.64^a^ | -1.25^bc^ | -3.66^c^ | -0.44^b^ | -1.93^bc^ | 0.465 | 0.0021 | <0.0001 | <0.0001 |
|  | *Peptococcus* | 1.15^b^ | 1.72^b^ | 1.31^b^ | 4.21^a^ | 1.16^b^ | -0.14^c^ | 0.232 | <0.0001 | <0.0001 | <0.0001 |
|  | *Peptostreptococcus* | -1.80^b^ | 0.00^a^ | -1.72^b^ | -1.89^c^ | -2.16^d^ | -4.62^e^ | 0.018 | <0.0001 | <0.0001 | <0.0001 |
|  | *Phascolarctobacterium* | -0.12 | 0.01 | -0.04 | 0.63 | 0.12 | -0.52 | 0.041 | 0.7567 | 0.3575 | NA |
|  | *Romboutsia* | -0.03 | 0.00 | 0.05 | 0.00 | 0.07 | 0.00 | 0.023 | 0.0338 | 0.1923 | NA |
|  | Ruminococcaceae unclassified | -0.02^bc^ | 0.18^a^ | -0.08^c^ | 0.10^ab^ | -0.05^c^ | -0.29^d^ | 0.031 | 0.0738 | <0.0001 | <0.0001 |
|  | *Sellimonas* | 0.03^b^ | 0.15^a^ | -0.04^b^ | 0.03^b^ | 0.02^b^ | -0.17^c^ | 0.021 | 0.9647 | <0.0001 | <0.0001 |
|  | *Streptococcus* | -0.89^c^ | -1.16^c^ | 0.78^b^ | -1.21^c^ | 1.35^ab^ | 2.55^a^ | 0.325 | 0.2014 | <0.0001 | 0.0013 |
|  | *Terrisporobacter* | 0.00^b^ | -0.08^b^ | 0.00^b^ | 0.05^b^ | 0.00^b^ | 0.31^a^ | 0.037 | 0.0075 | 0.0006 | 0.0006 |
|  | *Turicibacter* | 0.89^b^ | 0.08^c^ | 1.70^a^ | -0.02^c^ | 1.23^ab^ | 0.10^c^ | 0.146 | <0.0001 | 0.0658 | 0.0193 |
| Fusobacteriota |  | -2.74 | 0.04 | -1.04 | -0.83 | -0.68 | 0.06 | 0.983 | 0.1022 | 0.4936 | 0.3191 |
|  | *Cetobacterium* | -0.11^c^ | 0.00^b^ | 0.00^b^ | 0.00^b^ | 0.22^a^ | 0.00^b^ | 0.022 | 0.0266 | <0.0001 | <0.0001 |
|  | *Fusobacterium* | -0.93^c^ | 7.65^b^ | -3.18^c^ | 16.98^a^ | -0.44^c^ | -25.35^d^ | 0.966 | 0.0911 | <0.0001 | <0.0001 |
| Proteobacteria |  | 0.03 | 0.56 | 0.10 | -2.62 | 0.61 | 0.07 | 0.735 | 0.1107 | 0.051 | 0.0763 |
|  | *Anaerobiospirillum* | -0.01 | -0.03 | -0.07 | -0.05 | 0.02 | 0.02 | 0.024 | 0.9288 | 0.0191 | 0.6954 |
|  | *Escherichia-Shigella* | 0.02^b^ | -7.09^c^ | -0.05^b^ | -8.04^c^ | -0.02^b^ | 12.53^a^ | 0.906 | 0.1890 | <0.0001 | <0.0001 |
|  | *Morganella* | 0.00 | 0.22 | 0.00 | -0.15 | 0.00 | 0.02 | 0.015 | 0.6708 | 0.0323 | NA |
|  | *Parasutterella* | -0.22 | 0.59 | 0.05 | -0.64 | 0.51 | 1.08 | 0.297 | 0.3684 | 0.0107 | 0.0556 |
|  | *Proteus* | 0.00 | 0.45 | 0.00 | -0.49 | 0.00 | 0.11 | 0.048 | 0.6712 | 0.0326 | NA |
|  | *Sutterella* | 0.48^b^ | 0.36^b^ | 1.22^ab^ | 1.88^a^ | -1.20^c^ | -2.76^d^ | 0.219 | 0.0810 | <0.0001 | 0.0014 |

^1^ABX- = samples collected from dogs prior to antibiotic administration; ABX+ = samples collected after receiving metronidazole (20 mg/kg body weight twice daily) for two wk.
^2^SEM = pooled standard errors of the means.
^3^NA: data not normal, requiring non-parametric statistical analysis.
^a-e^Mean values within a row with unlike superscript letters differ using parametric analysis (P<0.05).

# **Supplemental Figure 1.** Unweighted (top) and weighted (bottom) principal coordinate analysis plot based on UniFrac distances of tubes containing cellulose.


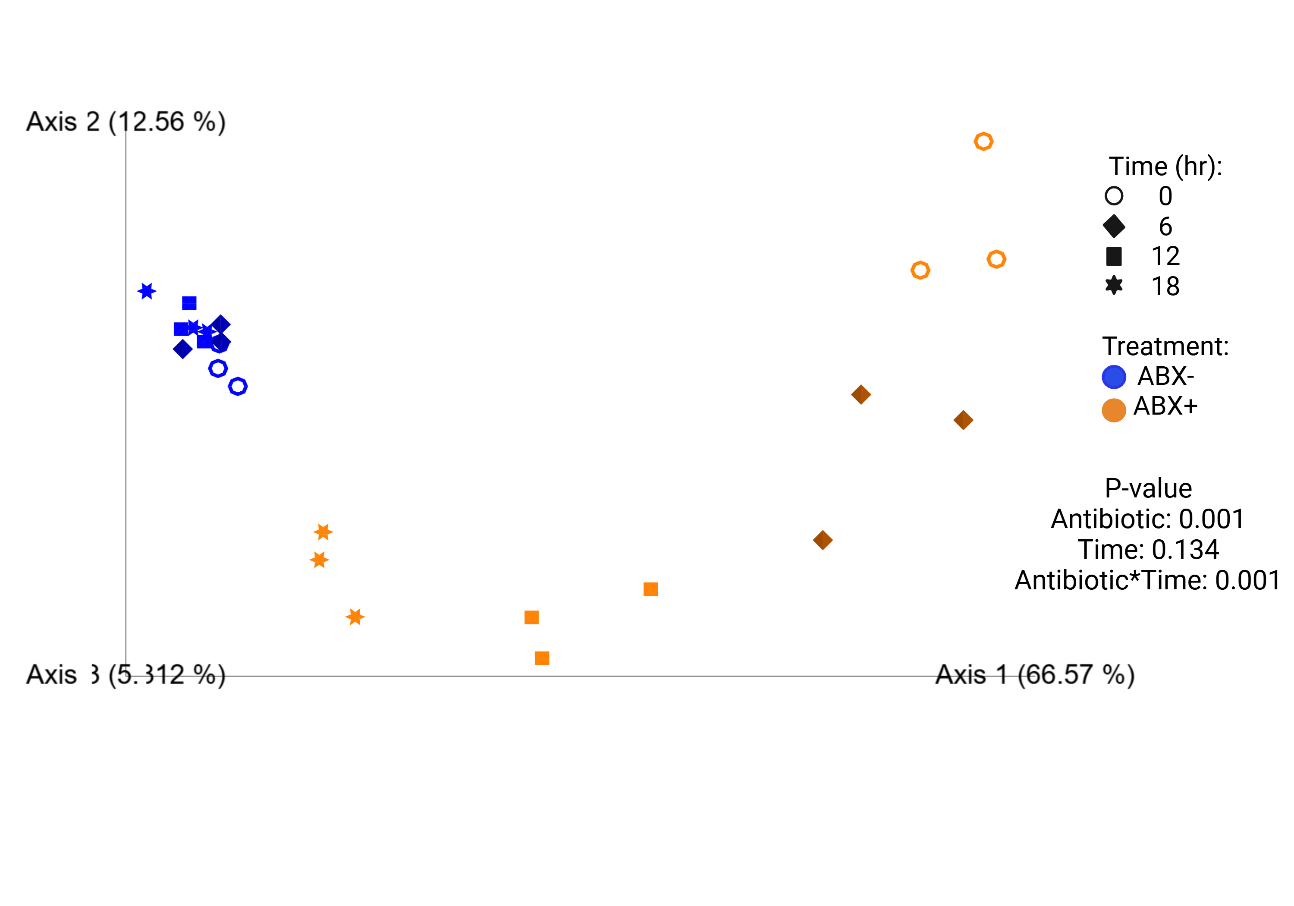


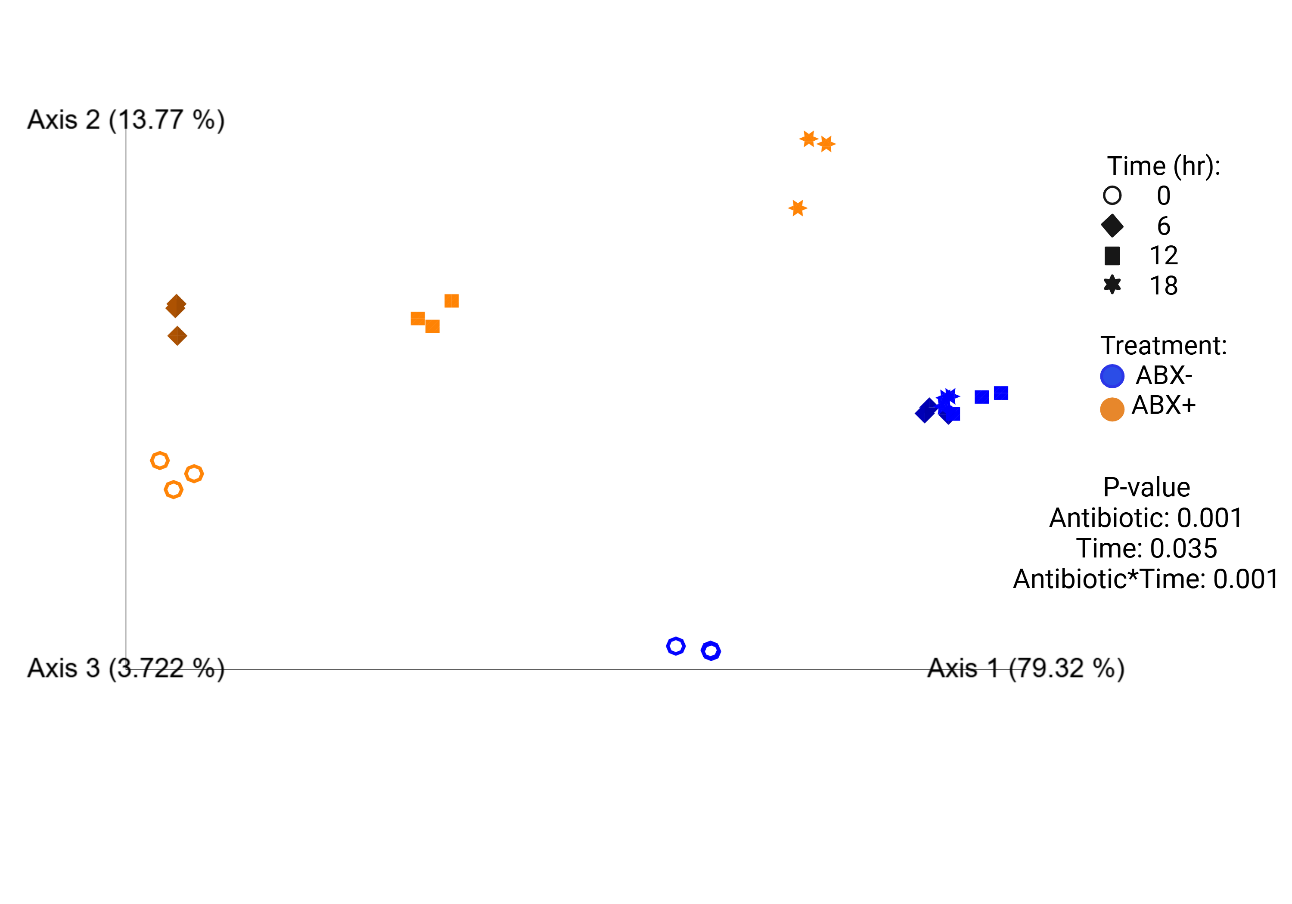

Supplement: Supplementary file 1 — Additional file 1. [file 42523_2025_459_MOESM1_ESM.docx]
